# α-Synuclein aggregation and brain atrophy in *SNCA*-A53T transgenic monkeys are correlated with parkinsonism

**DOI:** 10.1093/brain/awag046

**Published:** 2026-02-09

**Authors:** Jingkuan Wei, Shulin Li, Dingna Duan, Kunhua Wu, Xu Liu, Ran Zhu, Li Wang, Zhengwang Wu, Yu Kang, Chenyang Si, Hongjiang Zhang, Hong Wang, Yongchang Chen, Shaoxing Dai, Weizhi Ji, Gang Li, Lu Zhao, Yuyu Niu

**Affiliations:** State Key Laboratory of Primate Biomedical Research, Institute of Primate Translational Medicine, Kunming University of Science and Technology, Kunming, Yunnan 650500, China; Yunnan Key Laboratory of Primate Biomedical Research, Kunming, Yunnan 650500, China; State Key Laboratory of Primate Biomedical Research, Institute of Primate Translational Medicine, Kunming University of Science and Technology, Kunming, Yunnan 650500, China; Department of Radiology and BRIC, University of North Carolina at Chapel Hill, Chapel Hill, NC 27599, USA; Department of MRI, The First People’s Hospital of Yunnan Province, The Affiliated Hospital of Kunming University of Science and Technology, Kunming, Yunnan 650032, China; State Key Laboratory of Primate Biomedical Research, Institute of Primate Translational Medicine, Kunming University of Science and Technology, Kunming, Yunnan 650500, China; State Key Laboratory of Primate Biomedical Research, Institute of Primate Translational Medicine, Kunming University of Science and Technology, Kunming, Yunnan 650500, China; Department of Radiology and BRIC, University of North Carolina at Chapel Hill, Chapel Hill, NC 27599, USA; Department of Radiology and BRIC, University of North Carolina at Chapel Hill, Chapel Hill, NC 27599, USA; State Key Laboratory of Primate Biomedical Research, Institute of Primate Translational Medicine, Kunming University of Science and Technology, Kunming, Yunnan 650500, China; Yunnan Key Laboratory of Primate Biomedical Research, Kunming, Yunnan 650500, China; State Key Laboratory of Primate Biomedical Research, Institute of Primate Translational Medicine, Kunming University of Science and Technology, Kunming, Yunnan 650500, China; Yunnan Key Laboratory of Primate Biomedical Research, Kunming, Yunnan 650500, China; Department of MRI, The First People’s Hospital of Yunnan Province, The Affiliated Hospital of Kunming University of Science and Technology, Kunming, Yunnan 650032, China; State Key Laboratory of Primate Biomedical Research, Institute of Primate Translational Medicine, Kunming University of Science and Technology, Kunming, Yunnan 650500, China; Yunnan Key Laboratory of Primate Biomedical Research, Kunming, Yunnan 650500, China; State Key Laboratory of Primate Biomedical Research, Institute of Primate Translational Medicine, Kunming University of Science and Technology, Kunming, Yunnan 650500, China; Yunnan Key Laboratory of Primate Biomedical Research, Kunming, Yunnan 650500, China; State Key Laboratory of Primate Biomedical Research, Institute of Primate Translational Medicine, Kunming University of Science and Technology, Kunming, Yunnan 650500, China; Yunnan Key Laboratory of Primate Biomedical Research, Kunming, Yunnan 650500, China; State Key Laboratory of Primate Biomedical Research, Institute of Primate Translational Medicine, Kunming University of Science and Technology, Kunming, Yunnan 650500, China; Yunnan Key Laboratory of Primate Biomedical Research, Kunming, Yunnan 650500, China; Department of Radiology and BRIC, University of North Carolina at Chapel Hill, Chapel Hill, NC 27599, USA; State Key Laboratory of Primate Biomedical Research, Institute of Primate Translational Medicine, Kunming University of Science and Technology, Kunming, Yunnan 650500, China; Yunnan Key Laboratory of Primate Biomedical Research, Kunming, Yunnan 650500, China; State Key Laboratory of Primate Biomedical Research, Institute of Primate Translational Medicine, Kunming University of Science and Technology, Kunming, Yunnan 650500, China; Yunnan Key Laboratory of Primate Biomedical Research, Kunming, Yunnan 650500, China; Southwest United Graduate School, Kunming, Yunnan 650092, China

**Keywords:** Parkinson’s disease, transgenic monkeys, sleep disturbance, REM sleep behaviour disorder, MRI, brain structure

## Abstract

Mutations in the *SNCA* gene encoding α-synuclein underlie familial early-onset Parkinson’s disease. Pathological α-synuclein deposition may commence decades prior to the emergence of cardinal motor symptoms. Long-term investigation of brain and behavioural development in an *SNCA*-A53T transgenic macaque model offers critical insights into Parkinson’s disease progression.

In this study, we systematically characterized *SNCA*-A53T transgenic rhesus monkeys through multimodal assessments. Our results showed that these transgenic monkeys exhibited phosphorylated α-synuclein aggregation patterns and dopaminergic degeneration resembling Parkinson’s disease patients. Progressive motor and cognitive deficits were observed in transgenic monkeys with ageing. Polysomnographic analysis revealed rapid eye movement sleep behaviour disorder manifestations in transgenic animals. Four-year longitudinal MRI tracking demonstrated abnormal developmental patterns of cortical surface area alongside alterations in thickness and volume. The single-cell transcriptome revealed that astrocyte-specific gene dysregulation and cell loss contribute to brain atrophy in transgenic monkeys. Cortical and subcortical grey matter regions showing volume reduction were functionally associated with behavioural deficits and differentiated transgenic animals from wild-type controls.

Collectively, this comprehensive study provides evidence that *SNCA*-A53T transgenic monkeys recapitulate Parkinson’s disease pathophysiology while demonstrating the utility of longitudinal monitoring in genetically engineered non-human primates for tracking neurodegenerative disease progression.

## Teaser

Wei *et al.* show that *SNCA*-A53T transgenic monkeys recapitulate the natural progression of Parkinson’s disease. The animals display REM sleep behaviour disorder and region-specific brain atrophy as well as motor and cognitive deficits, supporting use of the model for studying early Parkinson’s disease pathophysiology.

## Introduction

Parkinson’s disease (PD) is a progressive neurodegenerative disorder characterized by the gradual loss of dopaminergic neurons and a reduction in dopamine transmission within the nigrostriatal pathway.^1^ Mutations in the *SNCA* gene, which encodes α-synuclein (α-syn), have been identified in familial PD cases with disease onset typically between 30 and 50 years of age, significantly earlier than in idiopathic PD.^2,3^ Patients carrying the A53T mutation in *SNCA* exhibit hallmark pathological features, including degeneration of nigrostriatal dopaminergic neurons and widespread aggregation of α-syn throughout the brain.^4^

Accumulating evidence suggests that nigrostriatal dopaminergic dysfunction and pathological α-syn deposition begin decades before the appearance of overt motor symptoms.^5,6^ This prodromal phase represents a critical window for early diagnosis, mechanistic investigation and therapeutic intervention.^7,8^ Retrospective clinical studies have identified several non-motor features as potential early-stage biomarkers, including hyposmia,^9,10^ depression,^11^ constipation^12,13^ and sleep disorders,^14-16^ along with subtle motor signs, such as rigidity, bradykinesia and postural instability, that precede the classical motor symptoms of PD.^5,17^ However, the specificity of most of these features is limited, because they are common in normal ageing and other neurodegenerative conditions, with rapid eye movement sleep behaviour disorder (RBD) being a notable exception with stronger predictive value.^18^

Numerous efforts have been made to develop experimental animal models that replicate the pathological and clinical features of PD.^19^ Toxin-induced models can produce rapid motor deficits,^20^ but they fail to recapitulate the gradual and multifaceted progression of PD, making them suboptimal for biomarker discovery. Transgenic (TG) mouse models overexpressing wild-type (WT) or mutant α-syn, in addition to models involving intracerebral or gastrointestinal injection of α-syn preformed fibrils, have shown promise for studying early-stage PD pathology. These models exhibit several PD relevant phenotypes, including olfactory impairment,^21^ gastrointestinal dysfunction,^22-24^ anxiety^25^ and sleep disturbances.^25^ Nevertheless, the translational relevance of these findings is limited by species-specific differences in neuroanatomy and physiology. Non-human primates, particularly macaques, offer a closer approximation to human neurobiology and are thus better suited to modelling the complex pathophysiological processes underlying PD. The macaque monkey, with its high degree of neuroanatomical and functional similarity to humans, provides a valuable platform for investigating early PD development and progression, yielding insights that are difficult to obtain from human studies alone.

Previously, we reported the successful generation of TG rhesus monkeys expressing the human A53T mutant α-syn (*SNCA*-A53T).^26^ In the present study, we conducted a longitudinal, multi-level investigation of the pathophysiological changes in these TG monkeys from birth to adulthood. Our earlier findings demonstrated pathological α-syn deposition in the postnatal brain from the time of birth. Building on this, we monitored PD-related phenotypes systematically over a 4-year period. Through integrated behavioural assessments, neuropathological examinations and neuroimaging analyses, we observed progressive α-syn aggregation, dopaminergic neurodegeneration and both motor and non-motor impairments. Notably, we identified RBD phenotypes and disruptions in sleep architecture, alongside symptom-associated regional brain atrophy as revealed by MRI. This longitudinal and multidimensional evaluation of the *SNCA*-A53T monkey model offers a comprehensive depiction of early PD development, providing valuable insights into the pathogenesis and progression of the disease.

## Materials and methods

### Ethics statement

The experimental plan was approved by the Institutional Animal Care and Use Committee of the State Key Laboratory of Primate Biomedical Research in advance (approval numbers K001111022-01 and LPBR202101009). All procedures involving animals were performed in accordance with the *Guide for the Care and Use of Laboratory Animals* (8th edition, US National Institutes of Health).

### Animals

The rhesus monkeys (*Macaca mulatta*) were housed and raised at the facility of the State Key Laboratory of Primate Biomedical Research. Eight TG monkeys and 15 age- and sex-matched WT monkeys were used in this study. Detailed information and involvement in the experiment for each animal are listed in Supplementary Table 1.

### Euthanasia and tissue preparation

Two TG monkeys and two WT controls (Supplementary Table 1) were euthanized using pentobarbital sodium (100 mg/kg, i.v.), then perfused with physiological saline. The brain tissue was collected with a surgical blade after confirmation of brain death.

The motor cortices of the monkeys were immediately frozen in liquid nitrogen and were stored at −80°C for further bulk RNA-sequencing (RNA-seq) and single-nucleus RNA-seq analysis. Notably, the motor cortices of two newborn A53T TG monkeys were also collected and stored at −80°C for bulk RNA-seq analysis. The brain tissues for histopathological analysis were fixed in 4% paraformaldehyde at 4°C for ≥24 h. After gradual dehydration in 20% and 30% sucrose solutions, the brain tissues were embedded in Tissue-Tek O.C.T. Compound (Sakura Finetek) and sectioned with a thickness of 20 μm using a Leica freezing microtome (CM 1860 UV).

### RNA sequencing

For single-nucleus RNA-seq, the nucleus was isolated using a nucleus isolation kit (SHBIO 52009-10) according to the instructions. snFAST-seq libraries were prepared using the SeekOne^®^ Single Cell Whole Trancriptome Kit according to the manufacturer’s instructions (SeekGene, Catalogue No. K00801). For bulk RNA-seq, total RNA was extracted according to the instruction manual for the TRlzol Reagent (Life Technologies). Sequencing libraries were generated using the Hieff NGS Ultima Dual-mode mRNA Library Prep Kit for Illumina [Yeasen Biotechnology (Shanghai) Co. Ltd.], following the manufacturer’s recommendations. Further details and analysis can be found in the Supplementary material, ‘Methods’ section.

### Histopathological analysis

#### Nissl stain

The frozen sections were put into 4% paraformaldehyde solution for 15 min, then washed with PBS three times (5 min each time) at room temperature. The sections were stained with Nissl staining solution (Catalogue No. E607316, Sangon Biotech) for 40 min, then dehydrated in an alcohol gradient, cleared with xylene and, finally, sealed with neutral resin.

#### Immunohistochemistry

The frozen sections were washed with PBS and blocked with 5% bovine serum albumin + 0.3% Triton X-100 in PBS for 2 h at room temperature. The tissues were incubated with primary antibodies overnight at 4°C. The primary antibodies used included anti-α-syn (ab212184, Abcam), anti-p-S129-α-syn (ab184674, Abcam) and anti-TH (AB152, Millipore). Subsequently, the sections were incubated with goat anti-mouse/rabbit HPR (PV-9000, ORIGENE). The DAB + substrate colour system (DAB-0031, MXB Biotechnologies) was added to visualize the target protein. The sections were dehydrated in an alcohol gradient, cleared with xylene and, finally, sealed with neutral resin.

#### Immunofluorescence

The frozen sections were washed with PBS and blocked with 1% bovine serum albumin + 0.3% Triton X-100 in PBS for 2 h at room temperature. The tissues were incubated with primary antibodies overnight at 4°C. The primary antibodies used included anti-p-S129-α-syn (ab59264, Abcam), anti-TH (MAB318, Millipore), anti-DAT (PA5-106751, Invitrogen) and anti-TUNEL (C1088, Beyotime). Subsequently, the sections were incubated with donkey anti-rabbit coupled with Alexa Fluor 594 (A32754, Invitrogen) or donkey anti-mouse IgG coupled with Alexa Fluor 488 (A21202, Invitrogen) for 3 h. The slices were sealed with fluorescent-shield mounting medium with DAPI (ab104139, Abcam).

#### Proximity ligation assay

The α-syn proximity ligation assay (PLA) experiments was conducted using Duolink^®^ kits supplied by Sigma-Aldrich as previously described.^27,28^ Briefly, the tissue sections were incubated with hydrogenase and blocked with Duolink^®^ block solution to dissolve non-specific binding sites after being treated with TritonX-100. Then, pre-prepared PLA pre-conjugated Syn211 antibodies (ab80627, Abcam) were added and incubated overnight at 4°C. After washing the sections with wash buffer and blocking endogenous peroxidase, ligation was performed at 37°C for 30 min, followed by amplification at 37°C for 2 h. The sections were then incubated with detection solution at room temperature for 1 h and, finally, with substrate solution (A, 1:70; B, 1:100; C, 1:100; and D, 1:50) at room temperature for 10 min. Subsequently, the sections were counterstained with haematoxylin, dehydrated through graded alcohols and xylene, and coverslipped with mounting media.

#### Imaging and image analysis

Nissl-stained and immunohistochemistry images were observed and photographed using the Olympus VS200 system (Olympus). Immunofluorescence images were acquired on a Leica SP8 confocal microscope (Leica). Images were acquired at identical settings (including laser power and digital gain/offset) to allow for comparisons of signal intensities between groups. Microscopic images were loaded in ImageJ v.2.14.0 (https://imagej.net/ij/) or OlyVIA XV Image Viewer 3 (Olympus) for analysis. The levels of total and phosphorylated α-syn (p-α-syn) in various brain regions were quantified as the optical density (OD) value of randomly selected regions of interest on the section, and six sections were selected and analysed from serial brain tissue sections at intervals of 200 μm from two animals for each group. The numbers of insoluble p-α-syn accumulations were counted in the whole substantia nigra (SN) or striatum (STr) regions on the section using the ‘Analyze Particles’ command of ImageJ, and 10 sections were selected and analysed from serial brain tissue sections at intervals of 200 μm from two animals for each group. The numbers of the Nissl^+^ or TH^+^ neurons were counted in the whole SN region on the section using Olympus OlyVIA, and 12 sections were selected and analysed from serial brain tissue sections at intervals of 200 μm from two animals for each group. The intensity of fluorescence was quantified as the average grey value of five randomly selected regions of interest on one section, and 12 sections were selected and analysed from serial brain tissue sections at intervals of 200 μm from two animals for each group.

### Nigrostriatal dopaminergic function

#### Single-photon emission computed tomography scanning

Single-photon emission computed tomography (SPECT) imaging using ^99m^Tc-TRODAT-1 to label dopamine transporter (DAT) was performed *in vivo*.^29^ The ^99m^Tc-TRODAT-1 was prepared from a lyophilized kit (Institute of Nuclear Medicine, Jiangsu) as previously reported by Bao *et al*.^30^ General anaesthesia of monkeys was induced by intramuscular injection of atropine (0.05 mg/kg) and ketamine (10 mg/kg) and maintained by intramuscular injection of zoletil/xylazine (6/2 mg/kg, with an additional dose of 2/0.67 mg/kg if needed). The animals were placed in a supine position with their heads fixed by a head holder in the SPECT scanner and were carefully monitored for physiological parameters throughout the scan. Oral administration of KClO_4_ (400 mg) for thyroid blockade was conducted 0.5 h before intravenous injection with 740 MBq (20 mCi) of ^99m^Tc-TRODAT-1. The brain SPECT scanning commenced 2 h later, using a dual-headed rotating gamma-camera (Infinia Hawkey, GE Healthcare) calibrated with a pixel matrix of 128 × 128 and energy window of 140 keV ± 20%. The duration of the SPECT imaging was 1.5 h, and the data were reconstructed by a back-projection algorithm with a Butterworth 3D post-filter and attenuation correction.

The regions of interest were drawn manually on the bilateral STr, including the caudate and putamen) and cerebellum (CB; serving as background area) in reference to the CT images. The analyst who drew regions of interest manually was blinded to the genotype. The striatal radioactivity uptakes were calculated by subtracting the mean counts per pixel in CB from the mean counts per pixel in STr in each hemisphere and dividing the result by the mean counts per pixel in the background: (STr − CB)/CB. The striatal radioactivity of each monkey was the average value of three consecutive layers. The measurement of SPECT data was performed using eFilm Workstation v.3.4 software (Merge Healthcare). To display the differential change between two sides, the side with lower radioactivity was defined as the affected side and the other was defined as the non-affected side. The striatal asymmetry index was calculated as (affected − non-affected)/[(affected + non-affected)/2].

### Sleep analysis

#### Surgery

TG and WT monkeys were surgically implanted with telemetry transmitters (TL11M2-D70-EEETM, DSI), as previously described.^31^ The EEG electrodes were placed on the cranium to monitor one standard bipolar derivation (FCz-FC3). The EMG electrodes were positioned parallel to the longitudinal axis of the neck muscles, with a distance of 10 mm between leads. Electrooculogram (EOG) leads were placed subcutaneously below the outer canthus and above the inner canthus of the right eye and sutured to the periosteum.

#### Data acquisition

A period of ≥2 weeks was allowed between the surgery and the start of electrophysiological recordings. The EEG, EOG and EMG signals and locomotor activity were recorded consecutively for 12 h in the dark cycle (light off) in the home cage of the monkeys. The telemetry signal was collected at a sampling rate of 500 Hz using the software DSI (Dataquest ART v.3.01, DSI) via a receiver (RMC-1, DSI). Further sleep stage identification and EEG spectral analysis can be found in the Supplementary material, ‘Methods’ section.

### MRI scanning and analysis

Magnetic resonance images were acquired using a 3.0 T clinical system (Achieva, Philips Healthcare). The sagittal 3D T_1_-weighted (T1w) images were acquired using an inversion-recovery prepared 3D spoiled gradient echo (SPGR) pulse sequence with a resolution of 0.375 mm × 0.375 mm × 0.5 mm. Diffusion-weighted images were acquired using 34 gradient directions with a resolution of 0.75 mm × 0.75 mm × 1.2 mm. Before the scan, all animals were pre-anaesthetized with atropine and ketamine (0.05/10 mg/kg, i.m.), which was maintained by intramuscular injection of zoletil/xylazine (6/2 mg/kg, with an additional dose of 2/0.67 mg/kg if needed). The animals were placed in a supine position with their heads fixed by a head holder in the MR scanner and were carefully monitored for physiological parameters throughout the imaging process.

The T1w MR images were processed in a double-blind manner using a computational pipeline developed in house.^32-34^ All T1w images were skull stripped, corrected for intensity inhomogeneity, and resampled to be an isotropic resolution (0.375 mm × 0.375 mm × 0.375 mm). Then, an algorithm based on machine learning was used to segment T1w images into grey matter (GM), white matter (WM) and CSF.^35^ Next, the inner cortical surface (GM–WM interface) and outer cortical surface (GM–CSF interface) of each brain hemisphere were reconstructed using a deformable surface method.^36^ The INIA19 macaque template (https://www.nitrc.org/projects/inia19/) was aligned non-linearly onto each image using diffeomorphic demons based on the tissue maps and, accordingly, its brain region labels were propagated onto each image and its corresponding cortical surfaces.^37^ For each subcortical, cortical or WM region, the volume was computed. For each cortical region, the total surface area and average cortical thickness were also computed. To study the vertex-wise maps of cortical thickness and surface area development, each inner surface was mapped onto a sphere and further aligned non-linearly onto the age-matched spherical template in 4D macaque surface atlases^32^ using spherical demons.^38^ Accordingly, cortical thickness maps were resampled, smoothed, and averaged for all subjects at the same age in the same group. The surface area and local gyrification index were also computed for each vertex in the resampled middle and outer cortical surfaces,^39^ respectively. The regional volume changes in a certain age were calculated as the percentage change = (regional GM volume in WT group minus regional GM volume in TG group)/regional GM volume in WT group × 100.

Diffusion-weighted MR images were processed by FSL^40^ in a double-blind manner. Specifically, we first corrected eddy current distortion and constructed diffusion tensors and computed the fractional anisotropy (FA) and mean diffusivity (MD) images. Next, for each scan, we aligned the b0 image onto the corresponding T1w image (with region labels) based on mutual information. Then we propagated the region labels in T1w images to the FA and MD images and computed the mean FA and MD values in each region accordingly.

Notably, given that previous studies have shown that the volume^41,42^ and diffusion parameters^43^ of brain structure are different between sexes throughout the postnatal brain development in human and non-human primates, the global and local brain structural parameters of females and males were analysed separately, and only data for female monkeys were plotted.

### Behavioural test

All six TG monkeys and six selected age- and sex-matched WT monkeys (Supplementary Table 1) were involved in the behavioural test. For all the behavioural tests, the behavioural protocols were performed and observed by trained technicians who were blinded to the genotypes of the monkeys. The weight and head circumference (HC) of monkeys were measured by one experienced operator. The HC was measured between the glabella and the occipital protruberance (the occipital–frontal circumference) with a fibreglass tape. Two experienced examiners rated independently for the PD score, and three for the sleep pattern rating scale. Data of the same test from different examiners were averaged before further analysis. The training and testing of fine motor test, object discrimination test and working memory test of all monkeys were distributed to two examiners. Further details can be found in the Supplementary material, ‘Methods’ section.

### Statistical analysis

The statistical analyses were performed with SPSS v.19.0 (SPSS, Chicago, IL, USA) or GraphPad Prism v.8 (GraphPad Software, Inc., San Diego, CA, USA), and graphs were plotted and constructed with GraphPad. Normality was tested using the Kolmogorov–Smirnov test, and the equality of variance was assessed using the *F*-test. Two sets of independent samples were compared using either two-tailed Student’s *t*-tests (normal distribution) or Mann–Whitney U-tests (non-normal distribution). Welch’s *t*-test were used instead of Student’s *t*-test when equal variance was not assumed. Multiple sets of measurements were analysed by one-way ANOVA (one independent variable) or two-way ANOVA (two factors) with Bonferroni’s *post hoc* test or by least significant difference method. The Pearson correlation between GM volume changes and ages was performed with SPSS. Based on GM volume data of nine brain subdivisions at 48 months, principal component analysis using the FactoMineR R package and discriminant analysis using SPSS software were applied to classify genotypes. Data were expressed as means ± standard error of the mean throughout, except in box-and-whisker plots, which show the median ± interquartile ranges, and minimum and maximum values. Sample sizes are reported in figure legends. Statistical significance was indicated as no significance (n.s.) *P* > 0.05, *P < 0.05, **P < 0.01, ***P < 0.001 or ****P < 0.0001.

## Results

### The pathological α-syn deposition in *SNCA*-A53T TG monkey brain

**Figure 1:**
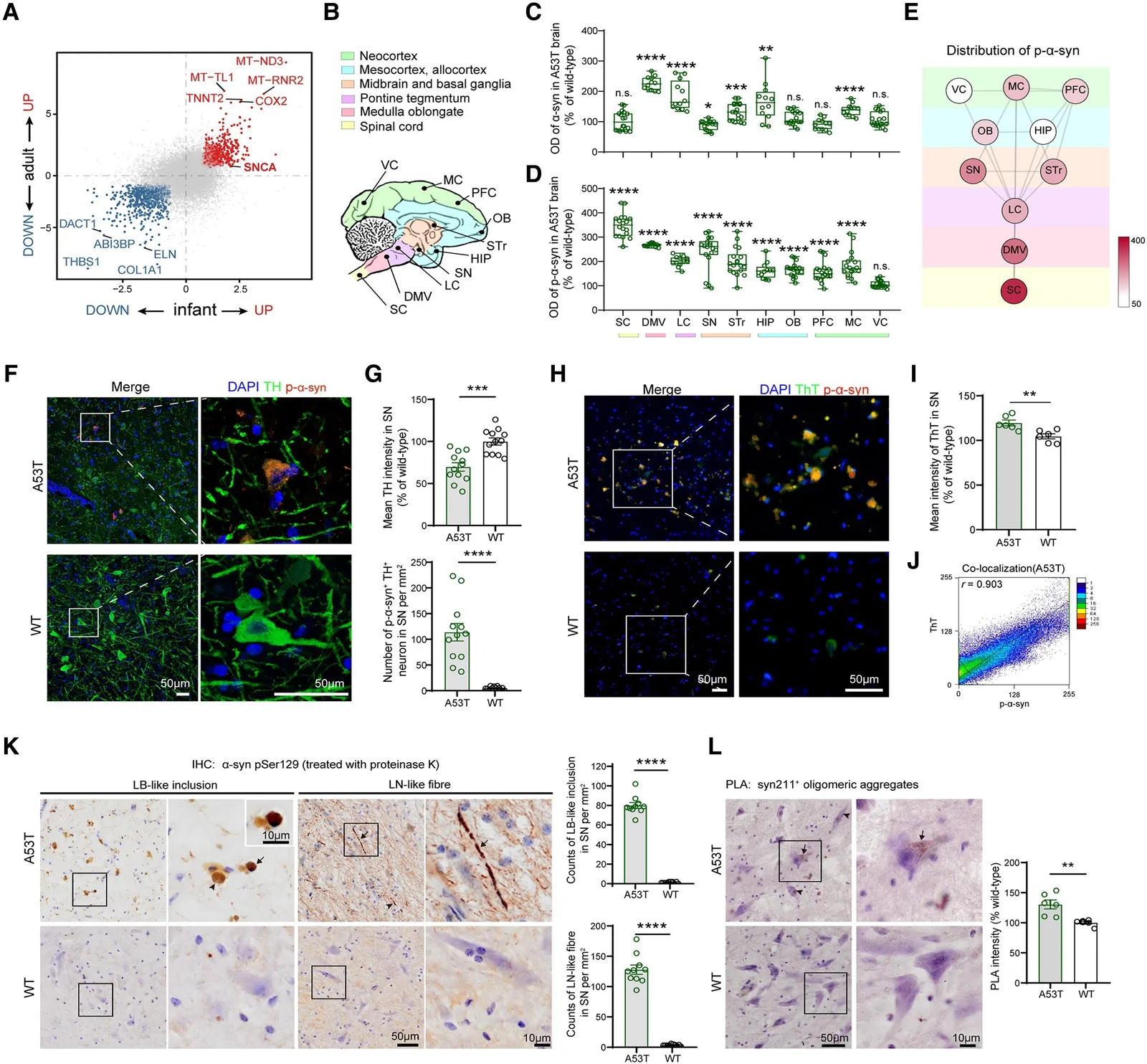
**Progressive synucleinopathy in A53T TG monkeys.** (**A**) Log_2_(fold change) between A53T and WT monkeys shows consistently high expression of *SNCA* between infant and adult brain tissue. (**B**) Synucleinopathy was tested in different regions of the monkey brain. (**C** and **D**) Quantifications of α-syn (**C**) and p-α-syn (**D**) immunoreactivity in different brain regions of A53T TG monkeys (*n* = 12 sections from two animals for each group; data for WT monkeys are shown in Supplementary Fig. 2A–D). Box edges delineate top and bottom quartiles, the centre line is the median, whiskers extend to minimum and maximum values, and circles depict individual data points. (**E**) Illustration showing the relationship of the extent of total p-α-syn pathology and the position of brain regions on the rostrocaudal axis for A53T TG monkeys. The colour of the circles corresponds to the intensity of immune staining according to **D**. The connecting line indicates the direct connections between brain regions. (**F**) Representative confocal images showing neurons in SN of A53T TG and WT monkeys, triple-labelled with DAPI (blue), p-α-syn (red) and TH (green). Scale bars = 50 µm. (**G**) TH intensity (*top*) and the number of p-α-syn^+^ TH^+^ neurons (*bottom*) is shown (*n =* 12 sections from two animals for each group). (**H**) Representative confocal images showing SN of A53T TG and WT monkeys, triple-labelled with DAPI (blue), p-α-syn (red) and ThT (green). Scale bars = 50 µm. (**I**) ThT intensity is shown (*n =* 6 sections from two animals for each group). (**J**) Co-localization analysis of p-α-syn and ThT. **(K)** Representative photomicrographs of insoluble p-α-syn (treatment with proteinase K) accumulation in SN of A53T TG monkey and WT monkey brains. The arrows and arrowheads indicate the structure of interest. The *right* panel shows a magnified view of the structure indicated by arrowheads in the *left* panel. Scale bars = 50 µm (*left*); 10 µm (*right*). Graphs show quantification of insoluble p-α-syn structures (*n* = 10 sections from two animals for each group). (**L**) Representative photomicrographs of α-syn oligomer (indicated by PLA signal) in SN of A53T TG monkey and WT monkey brains. Graph shows quantification of PLA signal intensity. Data are represented as the mean ± standard error of the mean, with circles denoting individual data points. One-way ANOVAs were used for multiple comparison in **C** and **D**. Pearson’s correlation analysis was used to assess co-localization (**J**). Student’s two-tailed *t*-tests were used when equal variance was assumed (**I**); otherwise, Welch’s *t*-tests were used for **G**, **K** and **L**. ^n.s.^*P* > 0.05, *P < 0.05, **P < 0.01, ***P < 0.001 or ****P < 0.0001. DMV = dorsal motor nucleus of the vagus; HIP = hippocampus; LC = locus coeruleus; MC = motor cortex; OB = olfactory bulb; p-α-syn = phosphorylated α-synuclein; PFC = prefrontal cortex; PLA = proximity ligation assay; SC = spinal cord; SN = substantia nigra; STR = striatum; TG = transgenic; TH = tyrosine hydroxylase; ThT = Thioflavin T; VC = visual cortex; WT = wild-type.

Eight *SNCA-A53T* TG (A53T TG) monkeys were generated previously, six of which survived to adulthood^26^ (Supplementary Table 1). In this study, two middle-aged adult A53T TG monkeys (TG04 and TG05) were sacrificed for transcriptomic and histopathological analyses. Integrating brain tissue RNA-seq data from the two infant A53T TG monkeys (TG01 and TG02), differentially expressed gene analysis revealed upregulated *SNCA* in both infant and adult monkeys compared with WT controls (Fig. 1A and Supplementary Fig. 1), suggesting continuous high expression of α-syn throughout growth in A53T TG monkeys. Gene Ontology (GO) analysis showed that downregulated genes in infant and adult A53T TG monkeys were enriched in pathways related to trans-synaptic signalling and chemical synaptic transmission (Supplementary Fig. 1). Genes involved in the respiratory electron transport chain and ATP synthesis were upregulated in infants (Supplementary Fig. 1), potentially reflecting an adaptive response of developing neural tissues to the mitochondrial biotoxicity of *SNCA*-A53T.

We showed previously that the expression of α-syn was increased in substantia nigra and striatum, but not cortex, in infant A53T TG monkeys.^26^ Further tests for p-α-syn showed no signal in the striatum of infant A53T TG monkeys.^26^ Here, we mapped α-syn distribution histopathologically throughout the CNS of adult A53T TG monkeys (Fig. 1B). Total α-syn was significantly elevated in multiple subregions of the spinal cord, brainstem and cerebral cortex of A53T TG monkeys relative to age- and sex-matched WT controls (Fig. 1C and Supplementary Fig. 2A and B; one-way ANOVA). Furthermore, quantified data revealed a marked increase in p-α-syn, a pathological isoform, within the spinal cord and various brain regions (Fig. 1D and Supplementary Fig. 2A and C; one-way ANOVA). Collectively, these findings indicate that sustained A53T mutant α-syn expression promotes pathological α-syn accumulation. Our histological examination revealed that total α-syn and p-α-syn deposition in affected brain regions exhibited a heterogeneous rostrocaudal distribution (Fig. 1E and Supplementary Fig. 2D). The p-α-syn pathology displayed a descending gradient from the spinal cord to the brainstem and the cortex, consistent with the Braak hypothesis of rostral-to-caudal synucleinopathy spread in PD patients.

Immunofluorescence also demonstrated that double-positive signals for p-α-syn and tyrosine hydroxylase (TH) were predominantly localized to the SN of A53T TG monkeys and reduced TH^+^ neurons in the SN of A53T TG monkeys (Fig. 1F and G; mean TH intensity was 69.66 ± 4.99 in A53T TG versus 100.0 ± 4.221 in WT, Welch’s *t*-test, *P* = 0.0001; the number of p-α-syn^+^ and TH^+^ neurons was 113.80 ± 17.16 in A53T TG versus 4.58 ± 0.92 in WT, Welch’s *t*-test, *P* < 0.0001). The p-α-syn puncta can also be labelled with Thioflavin T (ThT) (Fig. 1H–J; Student’s *t*-tests in Fig. 1I, *P* = 0.0058; Pearson correlation analysis in Fig. 1J), suggesting an amyloid-like structural feature of these aggregates. In the SN, insoluble p-α-syn aggregates resistant to proteinase K treatment were markedly increased, with some exhibiting morphologies resembling Lewy bodies or Lewy neurites (Fig. 1K and Supplementary Fig. 2E; the number of Lewy body-like inclusions was 80.00 ± 3.02 in A53T TG versus 1.20 ± 0.25 in WT, Welch’s *t*-test, *P* < 0.0001; the number of Lewy neurite-like fibres was 127.90 ± 7.59 in A53T TG versus 3.40 ± 0.45 in WT, Welch’s *t*-test, *P* < 0.0001). We also observed proteinase K-resistant diffuse plaques in the STr of A53T TG monkeys (Supplementary Fig. 3A). By measuring, Lewy neurite-like structures in SN were in an average length of 7.87 μm, and diffuse plaques in the STr had an average area of 809.7 μm^2^ (Supplementary Fig. 3B). Consistent with the increased insoluble α-syn, we also found enhanced intracellular PLA signal against α-syn oligomer in SN of A53T monkeys (Fig. 1L; Welch’s *t*-test, *P* = 0.0083). Along with synucleinopath (Supplementary Fig. 3C), an elevated level of p-Tau was also shown in A53T monkeys (Supplementary Fig. 3D), suggesting the co-occurrence of tauopathy with synucleinopathies, as reported previously for both A53T mutant mice and PD patients.^44^

### Nigrostriatal dopaminergic defects in A53T TG monkeys

**Figure 2:**
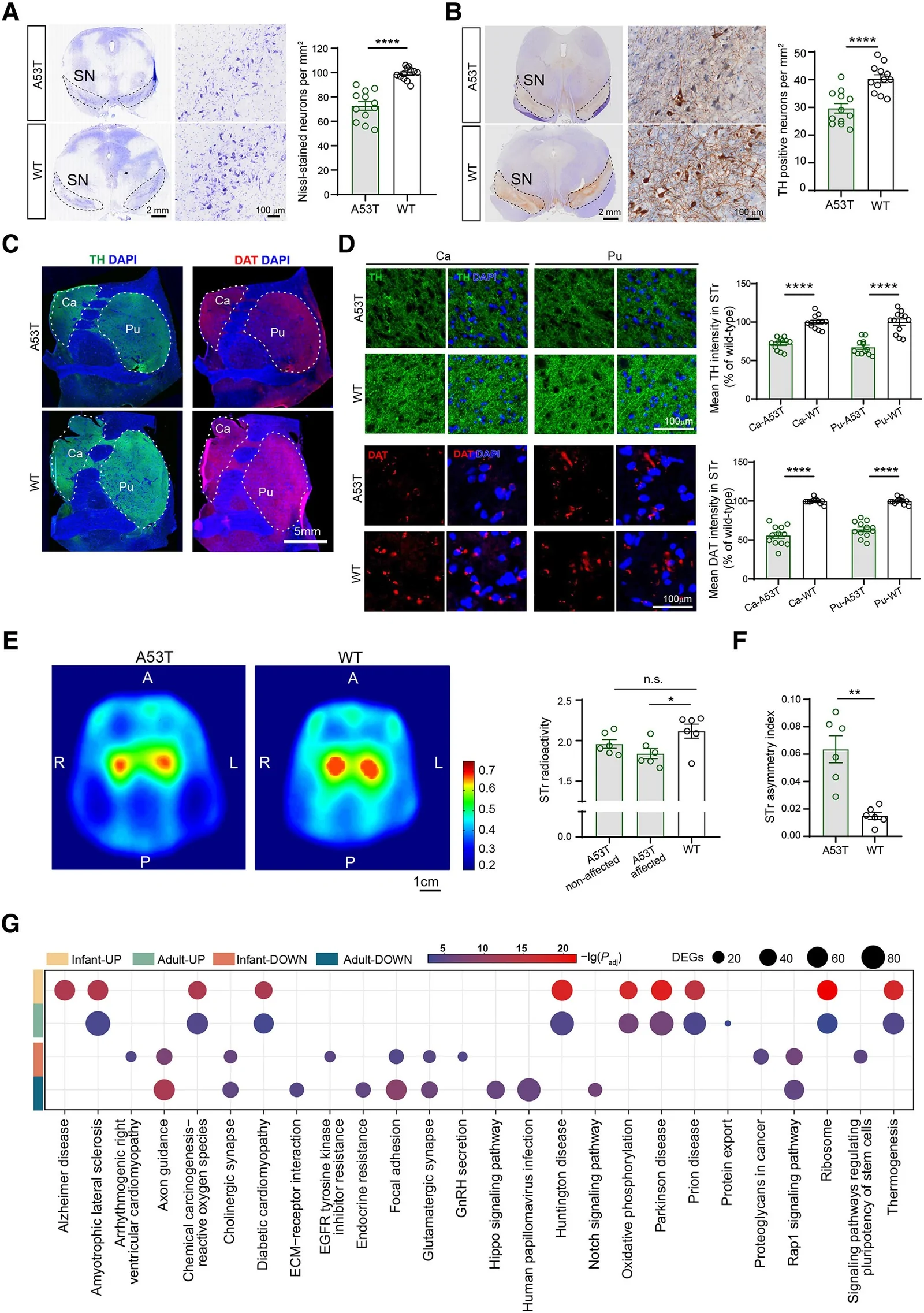
**Nigrostriatal dopaminergic defects in A53T TG monkeys.** (**A**) Representative micrographs of coronal mesencephalon sections with Nissl staining of A53T TG and WT monkey brains. Scale bars = 2 mm (*left*); 100 µm (*right*). Graph shows quantification of the number of Nissl-positive cells per millimetre squared in SN (*n* = 12 sections from two animals for each group). (**B**) Representative micrographs of coronal mesencephalon sections with TH staining of A53T TG and WT monkey brains. Scale bars = 2 mm (*left*); 100 µm (*right*). Graph shows quantification of the number of TH^+^ neurons per millimetre squared in SN (*n* = 12 sections from two animals for each group). (**C**) Representative confocal images showing striatum of A53T TG and WT monkeys double-labelled with DAPI (blue) and TH (green) in the *left* panel, and double-labelled with DAPI (blue) and DAT (red) in the *right* panel. Striatal regions are indicated by dashed line. Scale bar = 5 mm. (**D**) Representative confocal images showing caudate nucleus (Ca) and putamen (Pu) of striatum of A53T TG and WT monkeys double-labelled with DAPI (blue) and TH (green) on the *top* two rows, and with DAPI (blue) and DAT (red) on the *bottom* two rows. Scale bars = 100 µm. Graphs show quantification of TH (*top*) and DAT (*bottom*) intensity (*n* = 12 sections from two animals for each group). (**E**) Transverse SPECT neuroimaging showing the radioactivity signal of ^99m^Tc-TRODAT-1, a radiolabelled tropane that specific binds dopamine transporters (DAT) at the dorsal striatum. Representative images of the oldest A53T TG monkey (TG03, *left*) and the age-matched WT control (WT01, *right*) are shown. Colour bar indicates the intensity of radioactivity. Scale bar = 1 cm. Graph shows quantification of SPECT radioactivity. (**F**) Quantification of asymmetry index of A53T TG and WT monkeys (*n* = 6 animals for each group). (**G**) The top representative KEGG pathways of up- and downregulated genes in infant and adult monkey brain tissue. The Mann–Whitney U-test was used for the multiple comparison in **E**. Student’s two-tailed *t*-test was used when equal variance was assumed (**C**), otherwise Welch’s *t*-test (**A**, **B**, **D** and **F**). ^n.s.^*P* > 0.05, *P < 0.05, **P < 0.01, ****P < 0.0001. Details are provided in the ‘Materials and methods’ section. Ca = caudate nucleus; DAT = dopamine transporters; Pu = putamen; SN = substantia nigra; SPECT = single photon emission computed tomography; STr = striatum; TG = transgenic; TH = tyrosine hydroxylase; WT = wild-type.

Next, we examined whether PD-associated dopamine system degeneration occurred in the nigrostriatal pathway of A53T TG monkeys. Nissl staining indicated neuronal loss in SN of A53T TG monkeys (Fig. 2A; 72.67 ± 3.59 in A53T TG versus 99.08 ± 1.46 in WT, Welch’s *t*-test, *P* < 0.0001). Subsequently, immunohistochemistry for TH, a dopaminergic neuron marker, demonstrated that TH^+^ neurons were reduced to ∼26% of WT levels (Fig. 2B; 29.67 ± 1.73 in A53T TG versus 40.33 ± 1.44 in WT, Welch’s *t*-test, *P* < 0.0001). High-magnification imaging further revealed that TH^+^ neural fibres were fragmented in the SN of A53T TG monkeys. Furthermore, TUNEL staining showed increased apoptotic signal (Supplementary Fig. 4A), collectively suggesting selective neural cell loss in SN of A53T TG monkeys. Given the direct neural projections from the SN to the STr, the loss of TH^+^ neuronal soma in the SN was expected to reduce dopaminergic axons and terminals in the striatum. To confirm this, we examined TH and DAT expression immunohistologically in the sensorimotor STr and observed reduced immunoreactivity in both the caudate and putamen of A53T TG monkeys (Fig. 2C and D; Student’s *t*-tests for TH intensity comparison, *P* < 0.0001 for caudate and *P* < 0.0001 for putamen; Welch’s *t*-tests for DAT intensity comparison, *P* < 0.0001 for caudate and *P* < 0.0001 for putamen).

The radiolabelled tropane compound ^99m^Tc-TRODAT-1 specifically binds DAT in dopaminergic terminals and was used for *in vivo* SPECT imaging of nigrostriatal dopaminergic integrity. SPECT results revealed significantly lower bilateral DAT signals in all six adult A53T TG monkeys compared with WT controls (Fig. 2E; one-way ANOVA, A53T affected versus WT, *P* = 0.0315 and A53T non-affected versus WT, *P* = 0.2684). Given that the A53T TG monkeys were of heterogeneous ages during SPECT imaging, Pearson’s correlation analysis was applied to assess the relationship between DAT SPECT signals and age. Although a downward trend was observed, no significant linear correlation emerged between DAT SPECT signals and age in A53T TG monkeys (Supplementary Fig. 5A). Clinically, early-stage PD patients often exhibit asymmetric parkinsonism linked to hemispheric nigrostriatal dopaminergic degeneration. Accordingly, we found that the striatal asymmetry index was significantly elevated in A53T TG monkeys (Fig. 2F; Welch’s *t*-tests, *P* = 0.0037).

Impaired dopamine innervation was also supported by transcriptional profile analysis. The KEGG enrichment analysis uncovered a downregulation of synaptic transmission-related pathways and an upregulation of neurodegenerative disease-related pathways (Fig. 2H) in A53T TG monkey brains. Taken together, these results indicate an asymmetric and moderate impairment of dopaminergic pathways in the mesencephalon of A53T TG monkeys at young ages, suggesting that these monkeys are at the initial stage of the pathology.

### Subtle motor and cognitive defects in A53T TG monkeys

**Figure 3:**
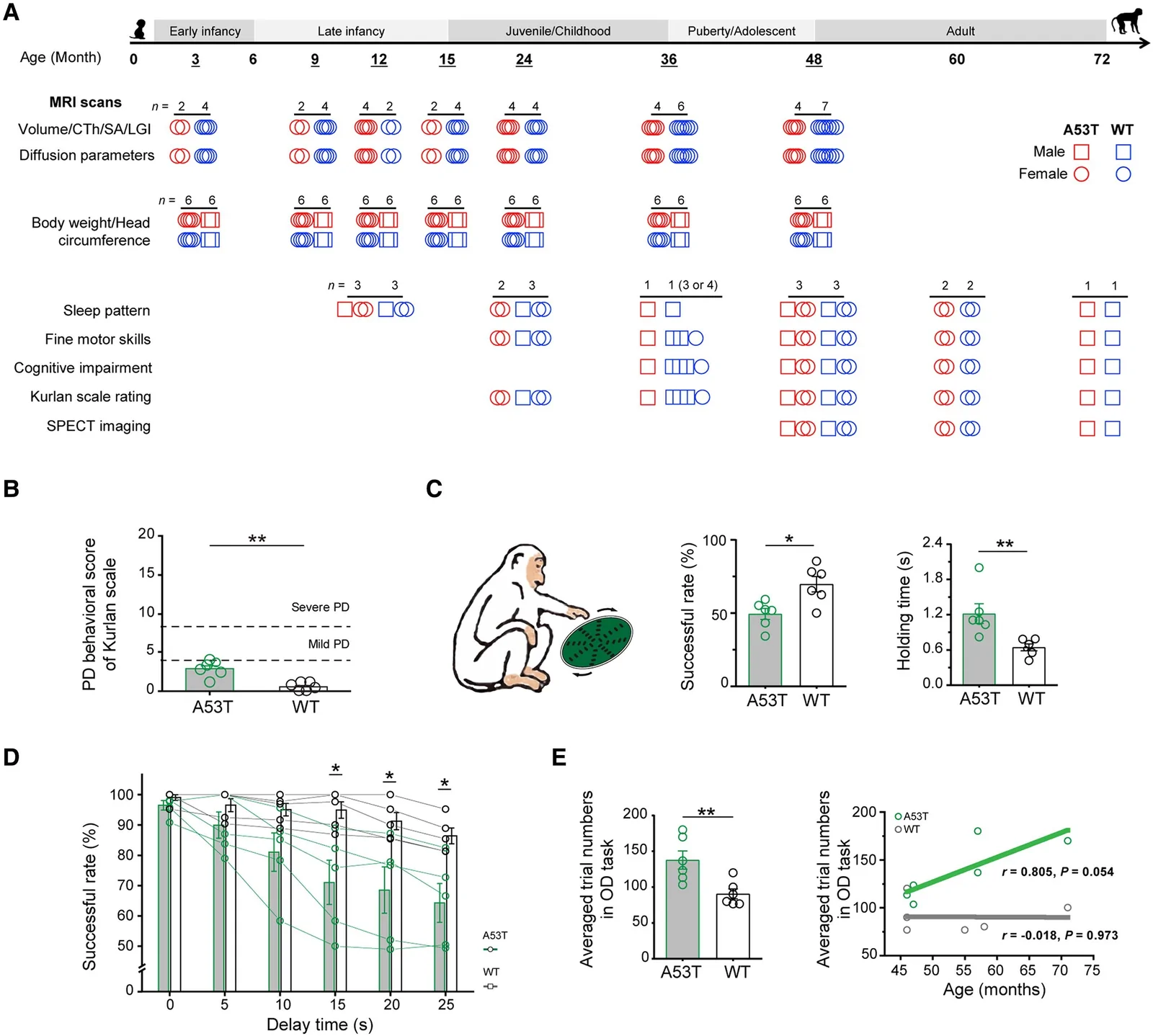
**Subtle motor defects and mild cognitive impairment in *SNCA-*A53T monkeys.** (**A**) Age distribution of A53T TG and WT monkeys when the MRI scans, general growth metrics and behavioural test were conducted. (**B**) Quantification of PD behavioural score. Dashed lines indicate the criteria for the diagnosis of severe PD and mild PD, respectively (*n* = 6 animals for each group, and for each animal the mean of six independent assessments is shown). (**C**) Sketch of the rotating Brinkman board test (*left*), and quantification of successful rate (*middle*) and holding time (*right*) of the test (*n* = 6 animals for each group, and for each animal the mean of five independent tests is shown). (**D**) The successful rate of A53T TG and WT monkeys at different delays in the delayed matching to sample task (*n* = 5 animals for each group). (**E**) Quantification of trial numbers for object discrimination (*left*) and the correlation of trial number and age (*right*) are shown (*n* = 6 animals for each group, and for each animal the mean of three independent tests is shown). Data are represented as the mean ± standard error of the mean, with the open circles denoting the mean of multiple tests of one animal. Student’s two-tailed *t*-tests were used when equal variance is assumed; otherwise, Welch’s *t*-test was used. *P < 0.05, **P < 0.01. CTh = cortical thickniss; LGI = local gyrification index; OD = object discrimination; PD = Parkinson's disease; SA = surface; SPECT = single photon emission computed tomography; TG = transgenic; WT = wild-type.

We assessed pathological changes comprehensively across physical and functional domains in A53T TG monkeys. A series of behavioural tests were conducted to evaluate parkinsonian phenotypes in A53T TG monkeys compared with age- and sex-matched WT controls (Fig. 3A and Supplementary Table 1). Initially, general growth metrics revealed comparable body weight and head circumference between A53T TG and WT monkeys during development (Supplementary Fig. 5B and C), indicating normal physical development in A53T TG monkeys from birth. Next, motor function was evaluated using the Kurlan scale,^45^ a modified version of the Unified Parkinson’s Disease Rating Scale (UPDRS) adapted for Old-World monkeys. A53T TG monkeys exhibited significantly higher PD-related motor scores than WT controls (Fig. 3B; Mann–Whitney U-test, *P* = 0.0065). Notably, there was only one monkey (TG04) that met diagnostic criteria for ‘mild PD’, suggesting that most A53T TG monkeys have not developed typical PD motor defects. Thus, more sensitive protocols are required to detect subtle behavioural changes in early PD stages.

The Brinkman board task^46^ was used to assess fine motor skills. A53T TG monkeys demonstrated greater difficulty in executing coordinated finger movements, resulting in significantly lower success rates and prolonged holding times compared with WT monkeys (Fig. 3C; Student’s *t*-tests, *P* = 0.0102 for successful rate, *P* = 0.0085 for holding time). However, Pearson’s correlation analysis revealed no significant linear relationship between these metrics and age in A53T TG monkeys (Supplementary Fig. 5D and E), suggesting that these subtle motor changes might not yet be correlated with pathological progression of PD in early stages.

To investigate non-motor deficits further, delayed matching-to-sample and object discrimination tasks were used. Delayed matching-to-sample performance declined progressively with increasing delay intervals in A53T TG monkeys (Fig. 3D), indicating impaired working memory. Object discrimination results revealed that A53T TG monkeys required more trials to complete tasks than WT controls, and Pearson’s correlation analysis confirmed an age-dependent worsening of performance (Fig. 3E; Student’s *t*-tests, *P* = 0.0085), consistent with progressive cognitive decline in A53T TG monkeys. To investigate whether there is neuronal loss in the motor or prefrontal cortex, we performed TUNEL staining but found no increased signals in these cortex regions of A53T monkeys compared with WT monkeys (Supplementary Fig. 4B and C). We speculate that in the early stage of PD, affected neurons have not yet undergone nuclear fragmentation but are already experiencing structural and functional impairments that might underlie the motor and working-memory phenotypes we observed.

### REM sleep behaviour disorder in A53T monkeys

**Figure 4:**
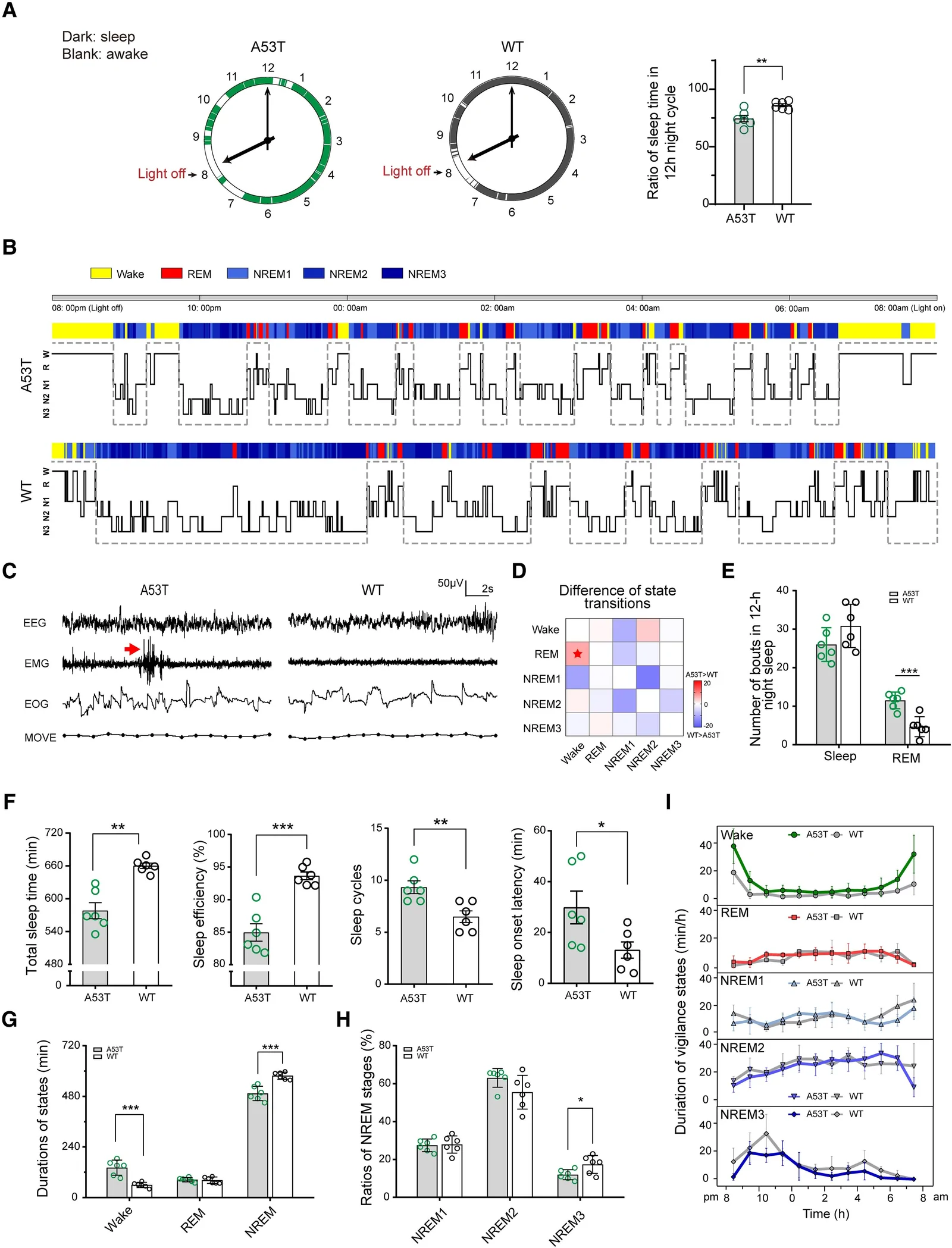
**Analyses of behavioural and polysomnographic recording reveal sleep disorder in A53T TG monkeys.** (**A**) Dial plates indicate sleep (dark) and awake (blank) of A53T (*left*) and WT (*right*) monkeys. The graph shows sleep time ratios in one 12 h nighttime from A53T TG and WT monkeys (*n* = 6 animals for each group, and for each animal the mean of five independent tests is shown). (**B**) Representative polysomnograms showing a 12 h night sleep of A53T TG (*top*) and WT (*bottom*) monkeys. (**C**) Representative traces of EEG, EMG, electrooculogram (EOG) and movement for A53T TG monkeys (*left*) and WT monkeys (*right*) in the REM sleep stage. Arrow indicates transient increase of EMG activity. (**D**) A heat map showing the differences of state transition during 12 h night sleep between A53T TG and WT monkeys. (**E**) Quantification of total bouts in 12 h night sleep and bouts in REM sleep of A53T TG and WT monkeys. (**F**) Quantifications of total sleep time, sleep efficiency, number of sleep cycles and latency of sleep onset in A53T TG and WT monkeys. (**G** and **H**) Quantifications of duration of vigilance states (**G**) and ratios of NREM stages (**H**) in A53T TG and WT monkeys. (**I**) Temporal distribution of vigilance states in A53T TG and WT monkeys. *P < 0.05, **P < 0.01, ***P < 0.001. NREM = non-rapid eye movement; REM = rapid eye movement; TG = transgenic; WT = wild-type.

In video-based nocturnal behavioural assays, A53T TG monkeys exhibited sleep fragmentation at the stage of falling asleep and showed significantly shorter total sleep durations compared with WT controls (Fig. 4A; Student’s *t*-tests, *P* = 0.0043). This observation parallels RBD, a highly specific non-motor symptom in early-stage PD.^47^ To evaluate the sleep disturbance of A53T TG monkeys in detail, we conducted polysomnographic recording using implantable transmitters. Different vigilance states were identified based on the visual inspection of EEG, EMG and EOG signals (Fig. 4B and Supplementary Fig. 6A). In WT monkeys, REM sleep was characterized by high-amplitude EOG activity and minimal-to-absent EMG activity (Fig. 4C, right). In contrast, A53T TG monkeys displayed transient EMG bursts during REM sleep (Fig. 4C, left), indicative of REM sleep without atonia, a hallmark of RBD. These phasic EMG activities are likely to have precipitated muscle twitches or movements, disrupting sleep continuity. To illustrate this point, we quantified the transitions among different vigilance states and found more transitions from REM to wakefulness in A53T TG monkeys compared with WT monkeys (Fig. 4D and E; Student’s *t*-tests, *P* = 0.1280 for number of bouts in sleep and *P* = 0.0006 for number of bouts in REM).

To explore sleep architecture, 12 h nocturnal sleep parameters were analysed. A53T TG monkeys showed reduced total sleep time and sleep efficiency, alongside increased sleep cycle frequency and prolonged sleep onset latency (Fig. 4F; Student’s *t*-tests). Their sleep was characterized by extended wakefulness and reduced non-REM (NREM) stage 3 duration compared with WT controls (Fig. 4G and H; Student’s *t*-tests). Hourly vigilance state distributions demonstrated elevated awakenings during the initial and final 2 h of sleep in A53T TG monkeys (Fig. 4I, *top*). The WT monkeys predominantly entered NREM3 during the first half of sleep, whereas A53T TG monkeys exhibited reduced NREM3 duration throughout the night (Fig. 4I, *bottom*), indicating fragmented sleep continuity. Collectively, these findings suggest impaired sleep stability in A53T TG monkeys.

Furthermore, spectral analysis revealed detailed differences in EEG power across sleep stages and frequency bands (Supplementary Fig. 6C). These electrophysiological features might provide insights for diagnosing early-stage PD.

### Brain atrophy in A53T TG monkeys

**Figure 5:**
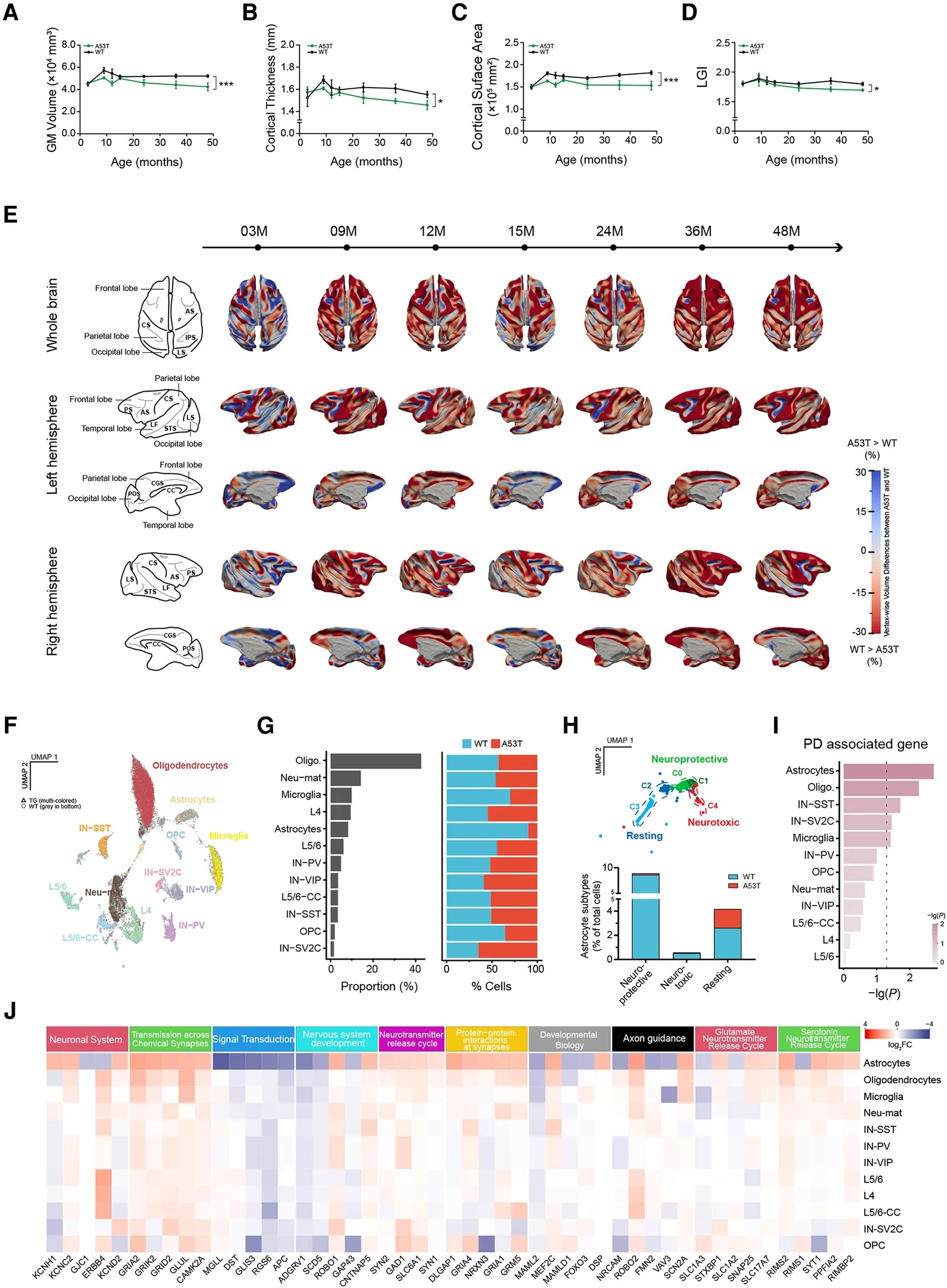
**Brain structural changes in TG monkeys and WT monkeys over time.** (**A**–**D**) The longitudinal brain changes by age for A53T TG and WT monkeys. The volume of grey matter (GM; **A**), cortical thickness (**B**), cortical surface area (**C**) and averaged local gyrification index (LGI; **D**) are shown (*n* = 4 animals for A53T TG; *n* = 7 animals for WT). (**E**) False-coloured cortical maps showing the difference in GM volume between TG and WT monkey brains. Top view of whole brain (*row 1*), lateral view of LH (*row 2*), medial view of LH (*row 3*), lateral view of RH (*row 4*) and medial view of RH (*row 5*) are shown with schematic diagrams on the *left* indicating the major lobes and sulci (*n* = 4 animals for A53T TG; *n* = 7 animals for WT). (**F**) Contribution of nuclei from A53T TG or WT monkeys to each cell type by single-nucleus RNA-sequencing analysis. Plot of A53T is multi-coloured accordingly; WT is coloured grey. *n* = 2 in each group. (**G**) Proportion of profiled nuclei per cell type (*left*) and proportion of different cell types per group (*right*). (**H**) The distributions of neuroprotective (cluster C0 and C1), resting (cluster C2 and C3) and neurotoxic (cluster C4) substate clusters in the astrocyte population (*top*) and proportions of each subtype (*bottom*) of A53T TG and WT monkeys. *n* = 2 in each group. (**I**) Parkinson’s disease-associated genes enriched for differentially expressed genes of different cell types. (**J**) Top reactome pathways enriched for differentially expressed genes of different cell types. Two-way ANOVA with Sidak’s *post hoc* test for **A**–**D**; *P_genotype_ < 0.05, **P_genotype_ < 0.01 and ***P_genotype_ < 0.001; spot with bars denotes mean ± standard error of the mean. AS = arcuate sulcus; CC = corpus callosum; CGS = cingulate sulcus; CS = central sulcus; IPS = intraparietal sulcus; LF = lateral fissure; LH = left hemisphere; LS = lunate sulcus; PD = Parkinson’s disease; POS = preoccipital sulcus; PS = principal sulcus; RH = right hemisphere; STS = superior temporal sulcus; TG = transgenic; WT = wild-type.

These transgenic monkeys provide a unique opportunity to investigate how sustained A53T mutant α-syn expression from birth to adulthood, alongside early pathological p-α-syn accumulation, impacts brain development. Longitudinal brain MRI scans were performed on A53T TG and WT monkeys at 3, 9, 12, 15, 24, 36 and 48 months of age (Fig. 5A and Supplementary Table 2). During development, WT brains exhibited rapid increases in GM volume, cortical thickness and cortical surface area until 15 months, followed by gradual decreases and stabilization from 15 to 48 months (Fig. 5A–C; two-way ANOVA). This trajectory is likely to reflect early-stage proliferation and synaptic pruning. A53T TG monkey brains showed reduced GM volume (*P* < 0.001), cortical thickness (*P* = 0.019) and cortical surface area (*P* < 0.001) but retained similar growth patterns. The local gyrification index, reflecting cortical folding complexity, demonstrated delayed maturation in A53T TG monkeys (Fig. 5D; two-way ANOVA, *P* = 0.026).

We also investigated the maturity of the nerve fibre tracts using diffusion tensor imaging. The FA and MD measurements revealed no differences in white matter maturity between A53T TG and WT monkeys (Supplementary Fig. 7A–C).

To illustrate better how the GM volume was affected in specific cortical regions of A53T TG monkeys, we coloured the cortical map according to the difference in GM volume between A53T TG and WT groups (Fig. 5E). At 3 months, A53T TG monkeys exhibited higher GM volume in the primary motor cortex (precentral gyrus), insular cortex and superior temporal gyrus compared with WT controls. However, these regions gradually atrophied after 9 months, and by 48 months, most cortical areas showed reduced GM volume in A53T TG monkeys except for the middle prefrontal gyrus and insular cortex. Similar trends were observed for cortical thickness and cortical surface area (Supplementary Fig. 7D and E).

To explore mechanisms underlying GM reduction, single-nucleus RNA-seq was performed on the primary motor cortex (Fig. 5F and Supplementary Fig. 8A). Clustering identified the same cell types in both A53T TG monkeys and WT controls, including neurons, astrocytes, oligodendrocytes and microglia (Fig. 5G, left). α-Syn expression was confirmed across all cell types, with notably higher levels in astrocytes and some neuronal subtypes (e.g. IN-IP, IN-PV and IN-SV2C) in A53T TG monkeys (Supplementary Fig. 8B). Single-cell transcriptomic studies of post-mortem brains of PD patients have suggested the proliferation of astrocytes in midbrain.^48,49^ However, similar studies in the prefrontal cortex show an unchanged or moderately decreased number of astrocytes.^50,51^ Here, we found that astrocytes were derived predominantly from WT monkeys (WT, 89.76% versus A53T TG, 10.24%; Fig. 5G, right), indicating profound astrocyte loss in A53T TG brains. Therefore, astrocyte loss could be an alteration during the early stage of PD, especially in prefrontal cortex. Further analysis shows that the proportion of neuroprotective substate astrocyte was reduced significantly (Fig. 5H and Supplementary Fig. 8D). Among all cell types, differentially expressed genes in astrocytes showed the strongest enrichment for PD-associated pathways (Fig. 5I and Supplementary Fig. 8C), suggesting heightened susceptibility to pathological p-α-syn. Furthermore, enrichment analysis revealed downregulated neural development pathways and upregulated synaptic transmission pathways in A53T TG astrocytes (Fig. 5J). Collectively, these single-cell transcriptomic data suggest that astrocyte gene dysregulation and subsequent cell loss contribute to brain atrophy in A53T TG monkeys.

### Region-specific brain atrophy linked to early parkinsonism in A53T TG monkeys

**Figure 6:**
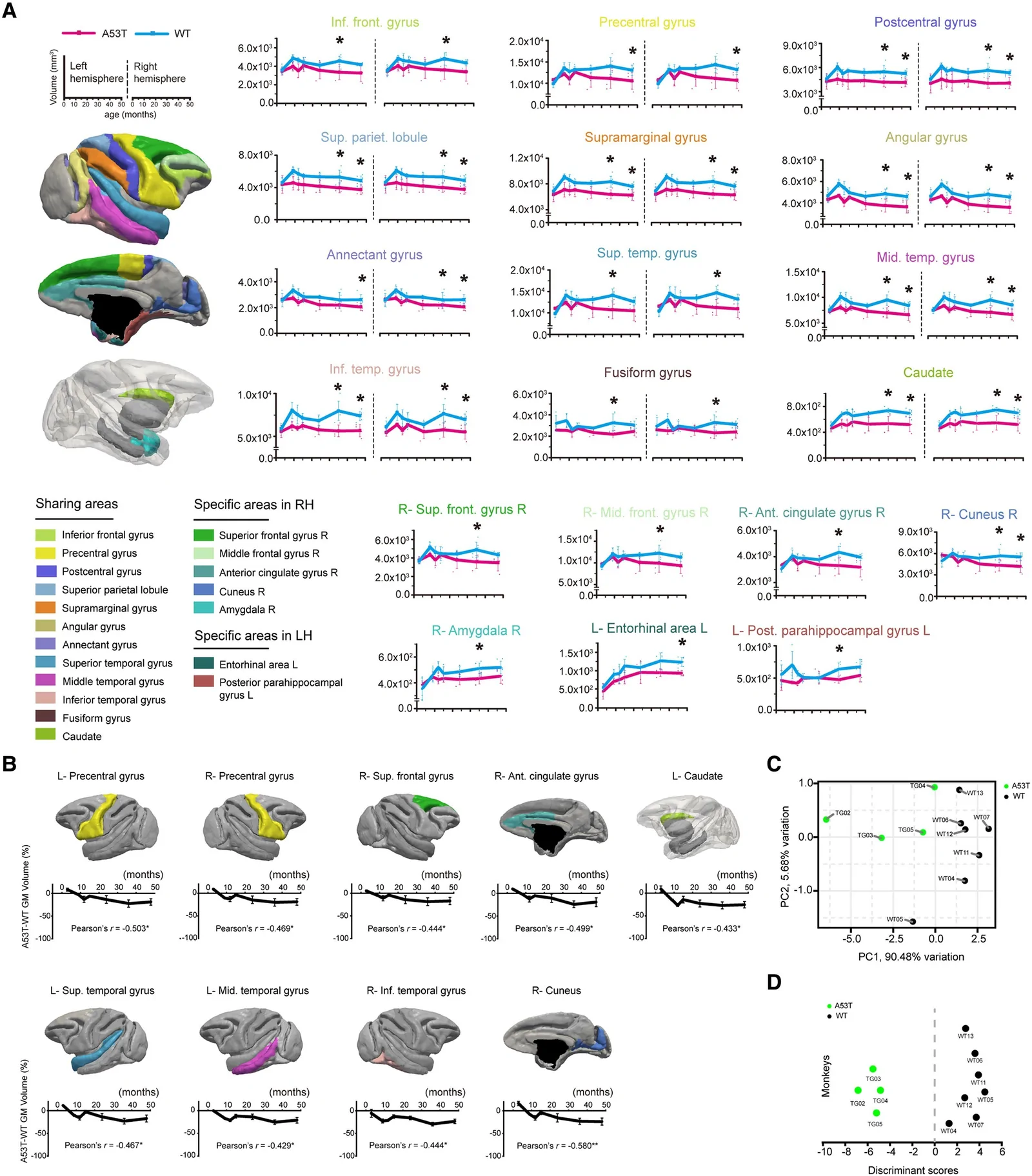
**Grey matter volume changes over time in brain subdivisions that are important for motor, cognitive and dopaminergic functions.** (**A**) GM volume of brain subdivisions for A53T TG and WT monkeys at different ages. Mann–Whitney U-test in all panels; *P < 0.025; spot with bars denotes the mean ± standard error of the mean. (**B**) Differences in GM volume between A53T TG and WT are plotted over time for brain subdivisions, with the brain maps (*top*) showing the location of the subdivisions. Pearson’s correlation between GM volume changes and ages was tested, and significance indicated as *P < 0.05 and **P < 0.01. (**C**) Principal component analysis using the significantly decreased volumes of the nine regions in the TG monkey brains. The *x*- and *y*-axes show principal component 1 (PC1) and principal component 2 (PC2), respectively. The contribution of each component to the total variance is indicated, and 90% of the variance can be explained by PC1. (**D**) Linear discriminant analysis using significantly decreased volumes of the nine regions in the TG monkey brains. The graphic represents the distribution of the samples considering their discriminant score. GM = grey matter; L = left; R = right; TG = transgenic; WT = wild-type.

Next, we analysed regional atrophy patterns in A53T TG monkey brains using the 4D macaque cortical surface atlas^32^ to explore preferential pathological p-α-syn deposition and elucidate relationships between localized atrophy and PD-associated motor/non-motor dysfunction. The GM volumes of 35 cortical subdivisions were compared between the A53T TG and WT monkeys. At 3 months, no significant differences in GM volume were observed in any subdivision (Supplementary Table 3). However, from 9 months onwards, A53T TG monkeys exhibited delayed GM volume growth in most subdivisions, with statistically significant deficits emerging in 19 regions by 36 or 48 months (Fig. 6A and Supplementary Table 3; Mann–Whitney U-test). Among these, 12 subdivisions showed bilateral symmetry, five were right hemisphere specific and two were left hemisphere specific.

To investigate further the significance of region-specific atrophy, we assessed whether the reduction in GM volume in specific brain regions was age dependent in A53T TG monkeys. Strikingly, nine brain subdivisions demonstrated reductions in GM volume significantly correlated with age in Pearson correlation analysis (Fig. 6B and Supplementary Table 3). These included the bilateral primary motor cortex (precentral gyrus) for voluntary movements^52^; the right superior frontal gyrus and the right anterior cingulate gyrus linked to attention and executive function^53,54^; the left caudate (part of the striatum) receiving dopaminergic inputs from the substantia nigra^55^; and three temporal gyri and the right cuneus gyrus (roles in PD pathology remain unclear). This regional atrophy pattern aligns with observed motor and cognitive deficits in A53T TG monkeys, suggesting a neuroanatomical basis for PD-related phenotypes.

Intriguingly, principal component analysis and discriminant analysis demonstrated clear separation between A53T TG and WT monkeys based on GM volume data from these nine subdivisions (Fig. 6C and D), highlighting their potential utility for early PD diagnosis.

## Discussion

In this study, we tracked the pathophysiological changes of A53T TG monkeys across multiple levels from birth over a 4-year period. Our data from the chronical and systematic investigation indicate that the A53T TG monkey replicates early-stage PD pathological changes and symptomatic procession. First, the A53T TG monkeys exhibited progressive α-syn aggregation and dopaminergic neuronal loss, which are hallmarks of canonical PD motor symptoms. These findings strongly suggest that the A53T TG monkey will develop overt PD in future as synucleinopathy and nigrostriatal dopaminergic deficits worsen. Furthermore, the rostrocaudal spreading of α-syn aggregation and asymmetric dopaminergic denervation observed in A53T TG monkey brains closely resemble the pathological changes in human PD, whereas few animal models have reproduced these phenotypes.^56,57^ Second, we identified a spectrum of non-motor symptoms, including mild cognitive impairment and RBD, whereas various rodent PD models have replicated only part of subsets or reproduced a different portfolio of these non-motor symptoms.^19^ Furthermore, we verified that the sleep disorder in A53T TG monkeys precisely mimics the RBD in humans, whereas rodent models show only RBD-like symptoms.^58,59^ Collectively, our longitudinal tracking from the birth of A53T TG monkeys outlines the dynamic trajectories of preclinical/prodromal PD pathology and sheds light on many new aspects of the early stage of this disease.

Insomnia affects the majority of PD patients, but evidence of its predictive value is relatively limited. There is convincing evidence that links sleep disorders to PD.^60^ RBD is a prodromal manifestation not only for PD but also for dementia with Lewy bodies^61^ and multiple system atrophy.^62^ We confirmed that the A53T TG monkeys experienced RBD, which is well acknowledged as a prodromal PD feature. In A53T TG monkeys at a young age, we unveiled the difficulty of initiating and maintaining sleep, with higher delta activity during NREMS, which might reflect increased sleep pressure. We think these findings provide mechanistic insights into insomnia–prodromal PD associations.

Advances in neuroimaging make it possible to assess several molecular mechanisms relevant to PD.^63^ Compared with healthy controls, volumetric analysis based on structural MRI scanning in PD patients has revealed GM and WM volume loss in various regions, including cortical areas of the frontal and temporal lobes,^64-66^ subcortical nuclei of the basal ganglia,^67-69^ in addition to the amygdala and hippocampus.^70,71^ Although it is convincing that clinically defined PD patients show specific patterns of brain atrophy, MRI studies of PD have reported inconsistent findings with respect to the present region and stage of cortical thinning and GM atrophy.^72,73^ One possible reason for these discrepancies could be different imaging protocols and analytical methods used in the literature for MRI measurement. The other possible reason could be the heterogeneity of subjects, which represents different disease durations, stages or clinical phenotypes in cross-sectional studies. To circumvent this problem, we scanned the brain of monkeys that bear the same genetic risk factor of PD longitudinally. Our results revealed GM atrophy and cortical thinning for A53T TG monkeys in the primary motor cortex (M1), prefrontal cortex, limbic system, caudate nucleus, inferior frontal gyrus, precentral gyrus, middle temporal gyrus and areas in the lateral parietal lobe. By correlating the morphometric features in the specific regions with age and symptoms, we illustrated that the brain atrophy progressed over time and underlined several early-stage symptoms. Furthermore, these brain morphometric features could be used to differentiate A53T TG monkeys from WT clearly.

## Conclusion

We propose that the *SNCA*-A53T TG monkey represents the first primate model that faithfully recapitulates the natural history of PD. Studies using this model can offer valuable insights into the initiation and progression of PD, with potential implications for both diagnosis and therapy. The longitudinal and multidimensional characterization of this model reveals new pathological features related to sleep behaviour and brain structure. We also suggest that integrating features derived from sleep patterns, EEG signals and MRI scans with established prodromal markers might significantly enhance the predictive power for PD onset.

## Supplementary Material

awag046_Supplementary_Data

## Data Availability

The data that support the findings of this study are available from the lead contact upon reasonable request.
