# Supplementary material for: α-Synuclein aggregation and brain atrophy in SNCA-A53T transgenic monkeys are correlated with parkinsonism
Source: Brain. 2026 Feb 9;149(9):3134–49. doi: 10.1093/brain/awag046 (PMC13548855; doi:10.1093/brain/awag046)
Supplement: Supplementary file 1 [file awag046_supplementary_data.pdf]

Supporting Information for

The α-synuclein aggregation and brain atrophy in *SNCA*-A53T transgenic monkeys correlate with parkinsonism

Jingkuan Wei[1,4,6], Shulin Li[1,6], Dingna Duan[2,6], Kunhua Wu[3,6], Xu Liu[1], Ran Zhu[1], Li Wang[2], Zhengwang Wu[2], Yu Kang[1,4], Chenyang Si[1,4], Hongjiang Zhang[3], Hong Wang[1,4], Yongchang Chen[1,4], Shaoxing Dai[1,4], Weizhi Ji[1,4,*], Gang Li[2,*], Lu Zhao[1,4,*], Yuyu Niu[1,4,5,7,*]

[1]State Key Laboratory of Primate Biomedical Research; Institute of Primate Translational Medicine, Kunming University of Science and Technology, Kunming, Yunnan, 650500, China

[2]Department of Radiology and BRIC, University of North Carolina at Chapel Hill, Chapel Hill, NC, 27599, USA

[3]Department of MRI, The First People's Hospital of Yunnan Province, The Affiliated Hospital of Kunming University of Science and Technology, Kunming, Yunnan, 650032, China

[4]Yunnan Key Laboratory of Primate Biomedical Research, Kunming, Yunnan, 650500, China

[5]Southwest United Graduate School, Kunming, Yunnan, 650092, China

[6]These authors contributed equally

[7]Lead contact

*Correspondence: wji@lpbr.cn (W.J.), gang_li@med.unc.edu (G.L.), zhaol@lpbr.cn (L.Z.), niuyy@lpbr.cn (Y.N.)

This PDF file includes:

Supplementary methods
Supplementary Figures 1 to 8
Supplementary Tables 1 to 3

Supplementary methods

Single-nuclei RNA Sequencing

Nuclei preparation The nucleus was isolated using a Nucleus Isolation Kit (SHBIO 52009-10) as instructions. RNase inhibitors (Sigma 3335399001) were added to the reagents before use. The samples were cut and transferred to a 5 mL tube containing lysate, mixed and lysed for 3 min on ice, then filtered through a 40μm cell filter (Sigma BAH136800040). Nucleus count and viability was estimated using fluorescence Cell Analyzer (Countstar® Rigel S2) with AO/PI reagent. Furthermore, the nucleuses were stained with 0.4% trypan blue and placed under a microscope. Subsequent experiments were performed if the nuclear envelope was intact and there were fewer impurities.

Single Nuclei Full-length RNA Sequence Transcriptome -seq (snFAST-seq) Library construction and sequencing snFAST-seq library were prepared using SeekOne® Single Cell Whole Trancriptome Kit according to manufacturer's instructions (SeekGene Catalog No.K00801). Briefly, an appropriate number of nuclei were mixed with reverse transcription reagents and added to the sample wells of the SeekOne® DD Chip S3. Then, Barcoded Hydrogel Beads (BHBs) and partitioning oil were dispensed into corresponding wells separately in Chip S3. Subsequently, Cell-containing reverse transcription reagents and BHBs were encapsulated into emulsion droplets using SeekOne® Digital Droplet System. Immediately following transferring emulsion droplets into PCR tubes, fifteen cycles of annealing (ramping from 8°C to 42°C) followed by a 5-min heat inactivation at 85°C were performed to obtain barcoded cDNA. Next, the barcoded cDNA was purified from broken droplet and then twice PCR reactions were performed to remove the majority of ribosomal and mitochondrial cDNA. AMPure beads were used to purify cDNA from the post PCR reaction mixture. Finally, one forth volume of cDNA was fragmented, end repaired, A-tailed and ligated into sequencing adaptor. DNA amplified by index PCR contains any part of polyA or non-PolyA RNA as well as Cell Barcode and Unique Molecular Index. The indexed sequencing libraries were purified using AMPure beads and quantified by quantitative PCR (KAPA Biosystems KK4824). The libraries were then sequenced on Illumina NovaSeq 6000 with PE150 read length.

Processing the Single Nucleus Full-length RNA sequencing data Fastp (version 0.22.0) was used to trim primer sequence and low-quality bases of raw reads and collect the basic

statistics. SeekSoul Tools (version 1.0.0) pipeline was used to process the cleaned reads and generated the transcript expression matrix. Normalization, scaling, dimensional reduction, clustering, and differential analysis were finished by Seurat (version 4.0.5). Finally, clusters were annotated to twelve major cell types based on per-cluster expression of a list of marker genes identified in a published human brain snRNA-seq dataset [1].

Bulk RNA Sequencing

RNA extraction Total RNA was extracted according to the instruction manual of the TRlzol Reagent (Life technologies, California, USA). RNA concentration and purity was measured using NanoDrop 2000 (Thermo Fisher Scientific, Wilmington, DE). RNA integrity was assessed using the RNA Nano 6000 Assay Kit of the Agilent Bioanalyzer 2100 system (Agilent Technologies, CA, USA).

Library construction and sequencing Sequencing libraries were generated using Hieff NGS Ultima Dual-mode mRNA Library Prep Kit for Illumina (Yeasen Biotechnology (Shanghai) Co., Ltd.) following manufacturer's recommendations. According to the manufacturer's instructions, an Illumina NovaSeq 6000 platform to generate PE-150 sequencing, and Illumina HiSeq 1500 system was used for SE-100 sequencing.

Data analysis Raw reads were first trimmed with fastp. Clean reads were mapped against the reference genome Mmul_10 with HISAT2 (version 2.2.1). Output SAM files were converted to BAM files using SAMtools (version 1.12). Following this step, a transcriptome for each sample was assembled using StringTie (version 2.1.7), and read counts were calculated by prepDE.py. DEGs of TG relative WT were compared using the DESeq2 (version 1.40.2), and genes with an adjusted P-value < 0.05 & $|\text{Fold Change}| \geq 1.5$ were assigned as differentially expressed.

Functional enrichment analysis Enrichment analysis of the differential genes was implemented by STRING and the R package ClusterProfiler (version 4.8.3). Pathway and process enrichment analysis was carried out with the following ontology sources: Gene Ontology (GO) Biological Processes, Kyoto Encyclopedia of Genes and Genomes (KEGG) Pathway, Reactome pathways [2]. In addition, we used clusterProfiler *enricher* function to test the statistical enrichment of PD-related genes from BrainBase database [3] in DEGs of each type cell.

Sleep analysis

Sleep stage identification Identification of the vigilance states in monkeys was performed over 30-s epochs using the NeuroScore software (NeuroScore 1.1, DSI, USA). Five different sleep states were identified, including wakefulness, rapid eye movement sleep (REM), non-rapid eye movement sleep stage1 (NREM1), non-rapid eye movement sleep stage 2 (NREM2) and non-rapid eye movement sleep stage 3 (NREM3) (Supplementary Fig. 5A). Wakefulness was characterized by low-amplitude, high-frequency EEG activity accompanied by high-amplitude EOG, EMG and movement activities. NREM1 was characterized by low-amplitude, mixed-frequency EEG accompanied by low EOG, EMG and movement activities. NREM2 and NREM3 were characterized by slow waves (high-amplitude, low-frequency waves: <2 Hz, >75 μV) appearing as >1/3 (NREM2) and >2/3 (NREM3) of an epoch on the EEG signal accompanied by very low EOG, EMG and movement activities. REM was characterized by low-voltage, mixed frequency activity on EEG accompanied by high-amplitude EOG activity, very low EMG activity and movement activities. In sleep cycle analysis, a sleep cycle consists of no less than 20 min of wakefulness and/or REM and no less than 20 min of NREMs. When the monkey in the wakefulness and/or REM state, once one consecutive NREMs over 5 min or a cumulative NREMs over 10 min occurred, it was considered that the monkey transited to NREMs, and vice versa.

EEG spectral analysis The EEG of artifact-free 30-s epochs was subjected to spectral analysis using Discrete Fourier Transform to calculate absolute power density between 1 and 60 Hz at 5 different sleep states. Spectral power computed as 10-min-bin that averaged from 30-s epochs over the full 12 hrs recording. Five EEG power of different frequency bands, including delta (1–4 Hz), theta (4–8 Hz), alpha (8–12 Hz), beta (12–30 Hz), gamma (30–60 Hz)] of 5 min typical states of wakefulness, REM and NREMs (NREM1, NREM2 and NREM3) were separately analyzed. Since the amplitude of the EEG raw data could influence the value of power in frequency bands, the normalized power and relative ratio were used to show the differences in spectral power between TG and WT. To calculate the normalised power, the amplitude of the TG and WT EEG data was normalized by mean of the absolute value of the WT amplitude, and then the EEG powers were transformed and compared. The heatmap of EEG spectrum of 5 min typical states of wakefulness, REM and NREMs (NREM1, NREM2 and NREM3) were plotted. Relative spectral activity of one sleep state was calculated as the ratio of one band in 5 frequency bands in TG or WT group.

Behavioral Test

Parkinsonism motor score All six TG monkeys and six selected age and sex paired WT monkeys (Supplementary table 1) were evaluated for PD motor symptoms 6 times (once a week) to get stable score. Monkeys were housed in an observation cage with enough room (1.2 m × 1.0 m × 1.0 m, length × width × depth) and a fixed bench, allowing monkeys to show a repertoire of behaviors, including turn around, rolling, walking with different gaits, et al. The front wall (1.2 m × 1.0 m) of the observation cage is made by glass, and monkey behaviors were video-recorded from the front wall without interruption for 60 min from 10:00 am to 11:00 am. Then the video was evaluated for the PD behavioral defects using scale modified from part A of the Kurlan scale which is the common rating scale of PD model in Old-World monkeys [4]. A sum-up score of (4-8) was defined as mild PD and (8-20) as severe PD.

Fine motor skills: A rotating Brinkman board (20 cm in diameter, clockwise at 12 rpm) containing 32 oval slots (15 mm × 8 mm × 6 mm, length × width × depth) were deployed to measure manual dexterity [5]. Candy balls with a round shape about 4 mm in diameter were placed in each slot as a reward. The TG monkeys and WT monkeys were trained to retrieve candy balls from the slots using their preferred and dominant hand in a training session for 3-10 days. The training session was ended when all 32 candies were taken out (motivation factor) and the total time of taking all candies out was no more than 6 min (execution factor). The test session conducted immediately following the training session in the next 5 consecutive days with video captured from the top. The number of candy balls successfully retrieved during first 30s and the candy holding time of first 20 successful take-ups (measuring frame by frame of the video sequence) were scored.

Object discrimination learning Object discrimination learning test was performed using the WGTA [6,7]. In the training session, there was only one well covered with a black lid in presentation board, and the monkey was trained to remove the lid of the well and take out the candy from the well. In our test part, to increase the challenge, there were 5 wells covered with different colors in the presentation board, only one well with pre-set color (i.e., green) lid contained a candy reward. Monkeys could choose one of the 5 wells to remove the lid to check for candy reward in the chosen boxes in one trial. The position of the pre-set color well switched randomly for each trial during the test. Once the success rate was over 85% in 20 consecutive trials, the test ended and the total trial number was counted. The maximum cutoff trials to stop one test and recording was 180 trials, if the successful rate could not be fulfilled. After one test, the pre-set color was reset to another (i.e. red) and a new test started. Three tests were conducted

for each monkey. The motivation factor criterion of this test was consecutive takeout of 30 candies, and the execution factor was that the complement of all 30 trials takes no more than 60 min (2 min per trial).

Working memory test The ability of working memory of the monkeys was assessed with a Delayed Response Task (Delayed matching to sample task). The VSDRT test used a Wisconsin General Test Apparatus (WGTA). In brief, the monkey was trained to remember the location of a peanut reward that was placed in one of two food wells on a tray. Then, both wells were covered with flat, gray plastic cards and a black opaque screen was lowered between the tray and the monkey. For a predefined delay period, the screen was raised and the test tray was shown to the monkey, allowing the monkey to displace one of the well covers (gray plastic card) to find whether there is the peanut reward. When the right well (with peanut) was chosen, it is recorded as a successful trial. In the training stage, monkeys need to complete sessions with five possible delay intervals (0, 5, 10, 15, 20 s) and 30 trials per session. The delay and well location pairings were varied pseudorandomly across trials such that each delay occurred six times in each testing session. Once the correct rate of the longest delay of the session was over 85%, a new session with longer delay intervals (5, 10, 15, 20, 25) was conducted, and so forth. Each animal was trained for 1000 trials to get the greatest delay intervals. In the test stage, the monkeys were tested the correct rate of all delay intervals they had been trained. In this study, only the data of delays intervals that both TG and WT monkeys involved was shown.

Night-sleep behaviors The Night-sleep behavior analysis were also done to compared sleep pattern and duration changes based on video-recordings [8]. Infrared video was captured for TG and WT monkeys living in cages without interruption from 19:00 to 9:00 am next day, and 12-hour recording from 20:00 to 8:00 am next day was analyzed. Three behavioral states, awake, sleep and bout from sleep, were distinguished and scored in 1 min epochs. In brief, animals were considered awake when locomotion occurred or when over 2 times of body or limb movement occurred within 1 min while in a seated or lying position. Falling into sleep was scored when the former 1 min was in awake state and had no body or limb movement occurred within 1 min while seated. Sleep maintenance was scored when the former 1 min was in sleep state and the body or limb movement occurred no more than twice within 1 min while seated.

Reference

1. Velmeshev, D., Schirmer, L., Jung, D., Haeussler, M., Perez, Y., Mayer, S., Bhaduri, A., Goyal, N., Rowitch, D.H., and Kriegstein, A.R. (2019). Single-cell genomics identifies cell type-specific molecular changes in autism. Science *364*, 685-689. 10.1126/science.aav8130.
2. Gillespie, M., Jassal, B., Stephan, R., Milacic, M., Rothfels, K., Senff-Ribeiro, A., Griss, J., Sevilla, C., Matthews, L., Gong, C., et al. (2022). The reactome pathway knowledgebase 2022. Nucleic Acids Res *50*, D687-D692. 10.1093/nar/gkab1028.
3. Liu, L., Zhang, Y., Niu, G., Li, Q., Li, Z., Zhu, T., Feng, C., Liu, X., Zhang, Y., Xu, T., et al. (2022). BrainBase: a curated knowledgebase for brain diseases. Nucleic Acids Res *50*, D1131-D1138. 10.1093/nar/gkab987.
4. Imbert, C., Bezard, E., Guitraud, S., Boraud, T., and Gross, C.E. (2000). Comparison of eight clinical rating scales used for the assessment of MPTP-induced parkinsonism in the Macaque monkey. Journal of neuroscience methods *96*, 71-76. 10.1016/s0165-0270(99)00184-3.
5. Chatagny, P., Badoud, S., Kaeser, M., Gindrat, A.D., Savidan, J., Fregosi, M., Moret, V., Roulin, C., Schmidlin, E., and Rouiller, E.M. (2013). Distinction between hand dominance and hand preference in primates: a behavioral investigation of manual dexterity in nonhuman primates (macaques) and human subjects. Brain Behav *3*, 575-595. 10.1002/brb3.160.
6. Nakamura, K. (2001). Perseverative errors in object discrimination learning by aged Japanese monkeys (Macaca fuscata). Journal of experimental psychology. Animal behavior processes *27*, 345-353.
7. Makori, N., Watson, R.E., Hogrefe, C.E., Lalayeva, N., and Oneda, S. (2013). Object discrimination and reversal learning in infant and juvenile non-human primates in a non-clinical laboratory. Journal of medical primatology *42*, 147-157. 10.1111/jmp.12041.
8. Chen, Y., Yu, J., Niu, Y., Qin, D., Liu, H., Li, G., Hu, Y., Wang, J., Lu, Y., Kang, Y., et al. (2017). Modeling Rett Syndrome Using TALEN-Edited MECP2 Mutant Cynomolgus Monkeys. Cell *169*, 945-955 e910. 10.1016/j.cell.2017.04.035.

Supplementary figures

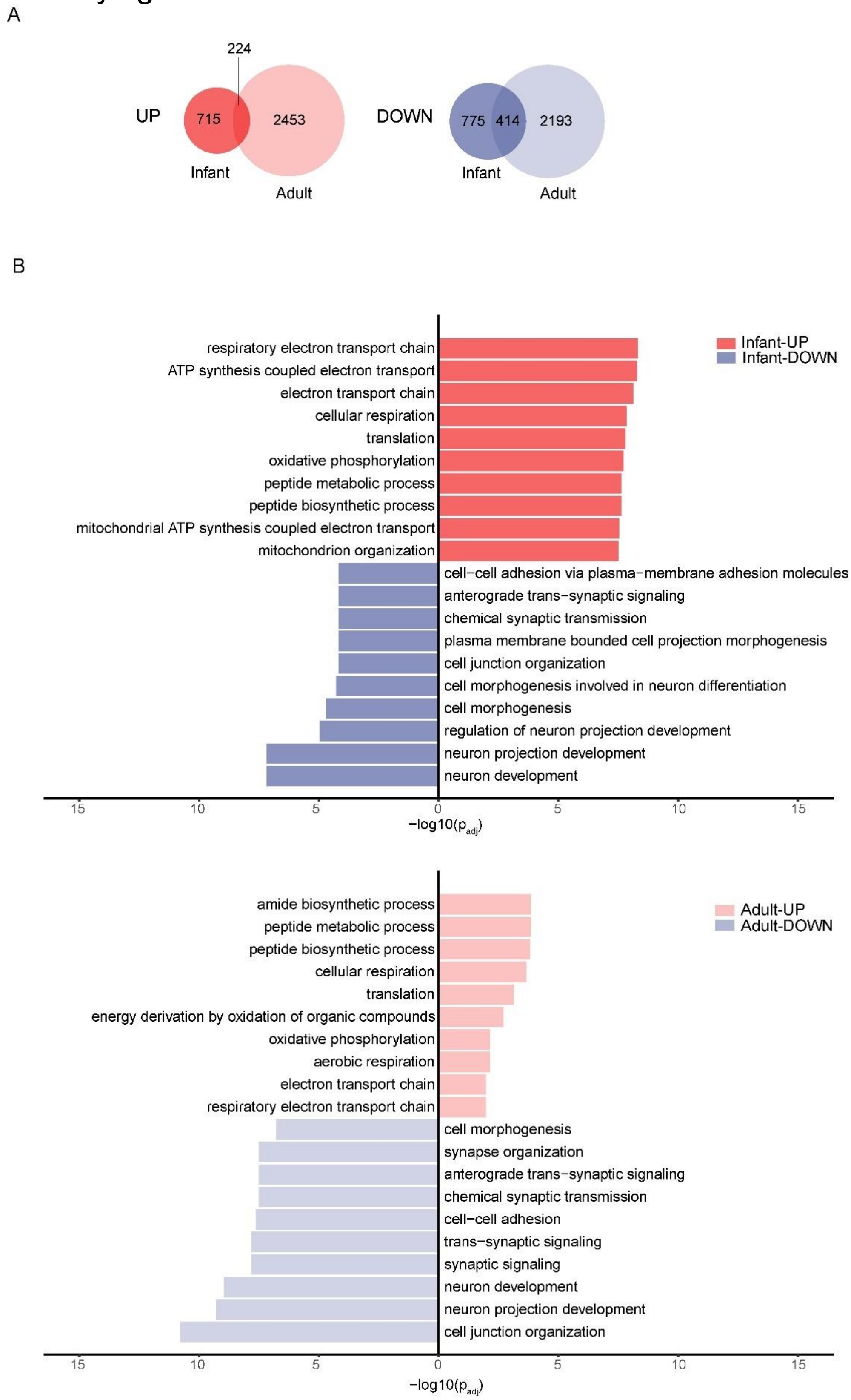

Supplementary Figure 1. Shared DEGs and pathways in infant and adult A53T TG monkeys compared with WT monkeys. (A) Overlap of DEGs between A53T and WT monkeys detected in infant and adult

monkey brain tissue. (B) GO biological process terms of the up-regulated and down-regulated genes in infant (top) and adult (bottom) monkey brain tissue.

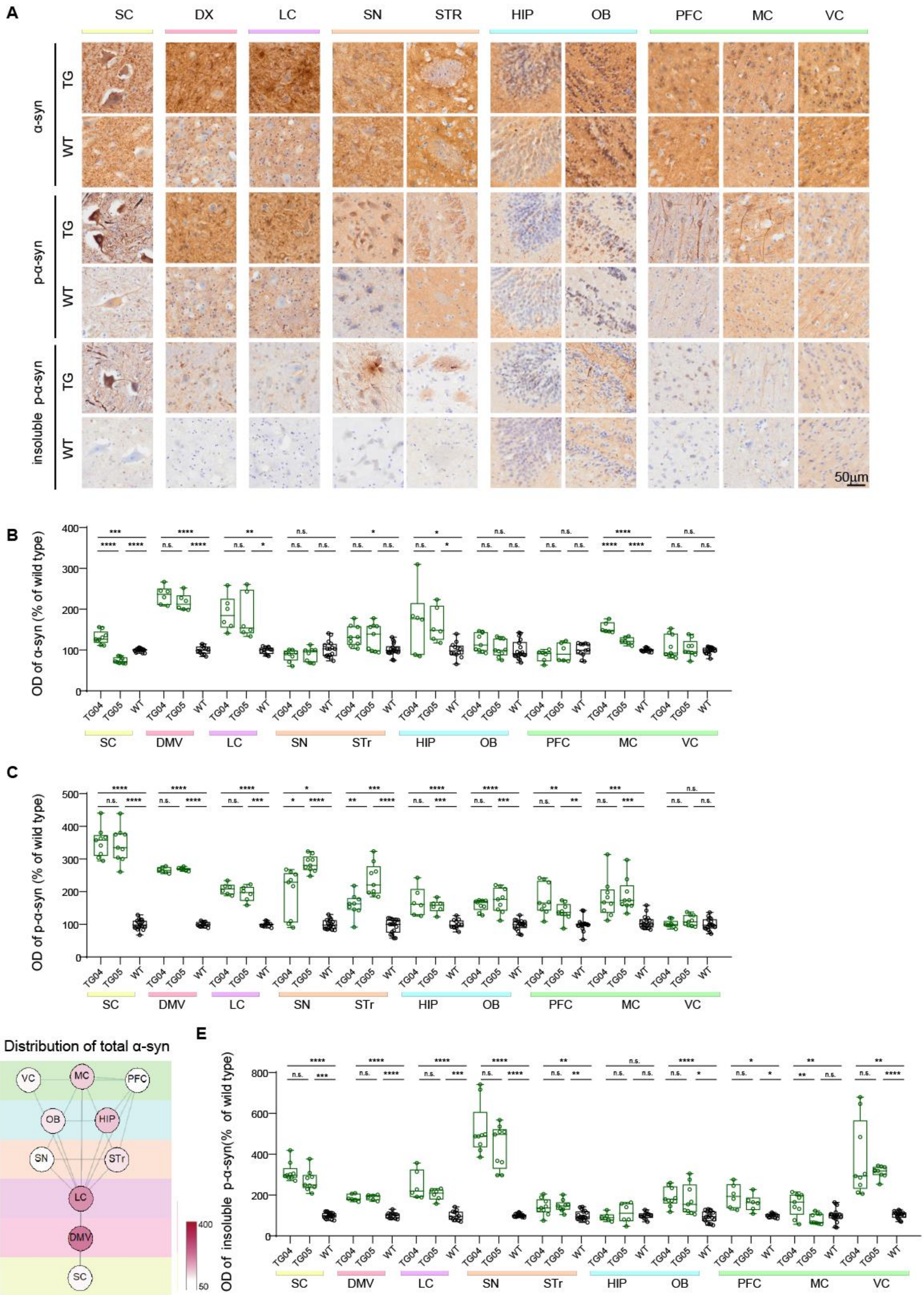

Supplementary Figure 2. Increased α-syn pathology in different brain regions. (A) Representative micrographs of α-syn and p-α-syn and insoluble p-α-syn (treatment with proteinase K) staining in different brain regions. (B, C and E) Quantifications of α-syn (B), p-α-syn (C) and insoluble p-α-syn (E)

levels in different brain regions. (D) Illustration showing the relationship of the extent of total α-syn pathology and the position of brain regions on the rostro-caudal axis for A53T TG monkeys. The color of the circles corresponds to the intensity of immune staining according to (Figure 1C). The connecting line indicates the direct connections between brain regions.

Box edges delineate top and bottom quartiles, the center line is the median, whiskers extend to minimum and maximum values and circles depict individual data points. n = 12 sections from 2 animals for each group. One-way ANOVA with Bonferroni's post hoc test and statistical significance indicated as $^{*}p < 0.05$, $^{**}p < 0.01$, $^{***}p < 0.001$, $^{****}p < 0.0001$, ns = not significant.

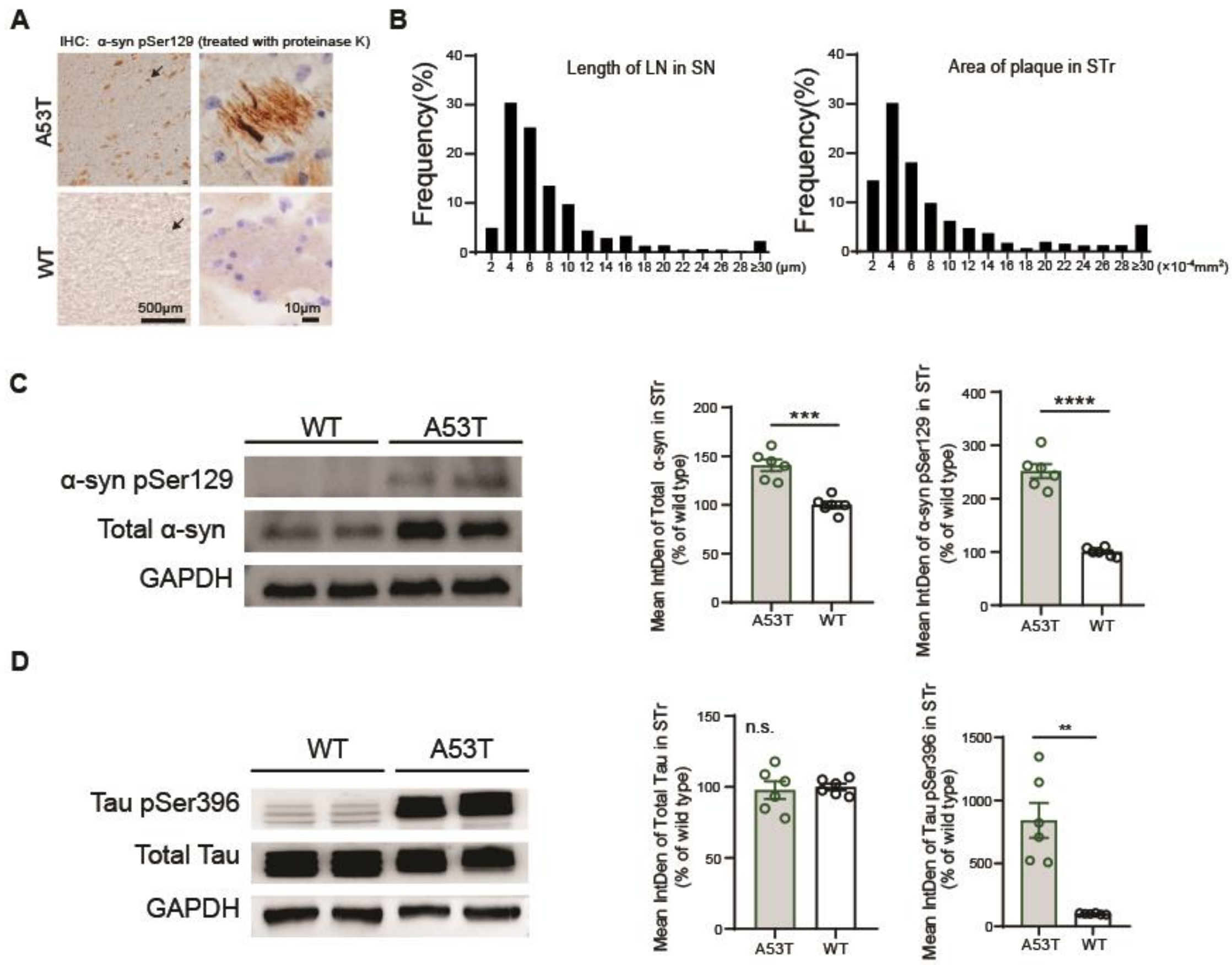

Supplementary Figure 3. Insoluble p-α-syn aggregation and tauopathy in A53T monkeys. (A) Representative photomicrographs of insoluble p-α-syn (treatment with proteinase K) accumulation in STr of A53T TG monkey and WT monkey brains. The arrows and indicate the structure in interest. The right panel show magnificent view of the structure indicated by arrowheads in left panel. Scale bars: 50 μm (left), 10 μm (right). (B) Distribution of length of LN-like fibers in SN (left) and area of plaques in STr (right) for A53T TG monkey. (C) Western Blot (left) and quantification (right) of p-α-syn and total α-syn. (D) Western Blot (left) and quantification (right) of p-Tau and total tau (n = 3 repeats from 2 animals for A53T monkey, n = 2 repeats from 3 animals for WT monkey).

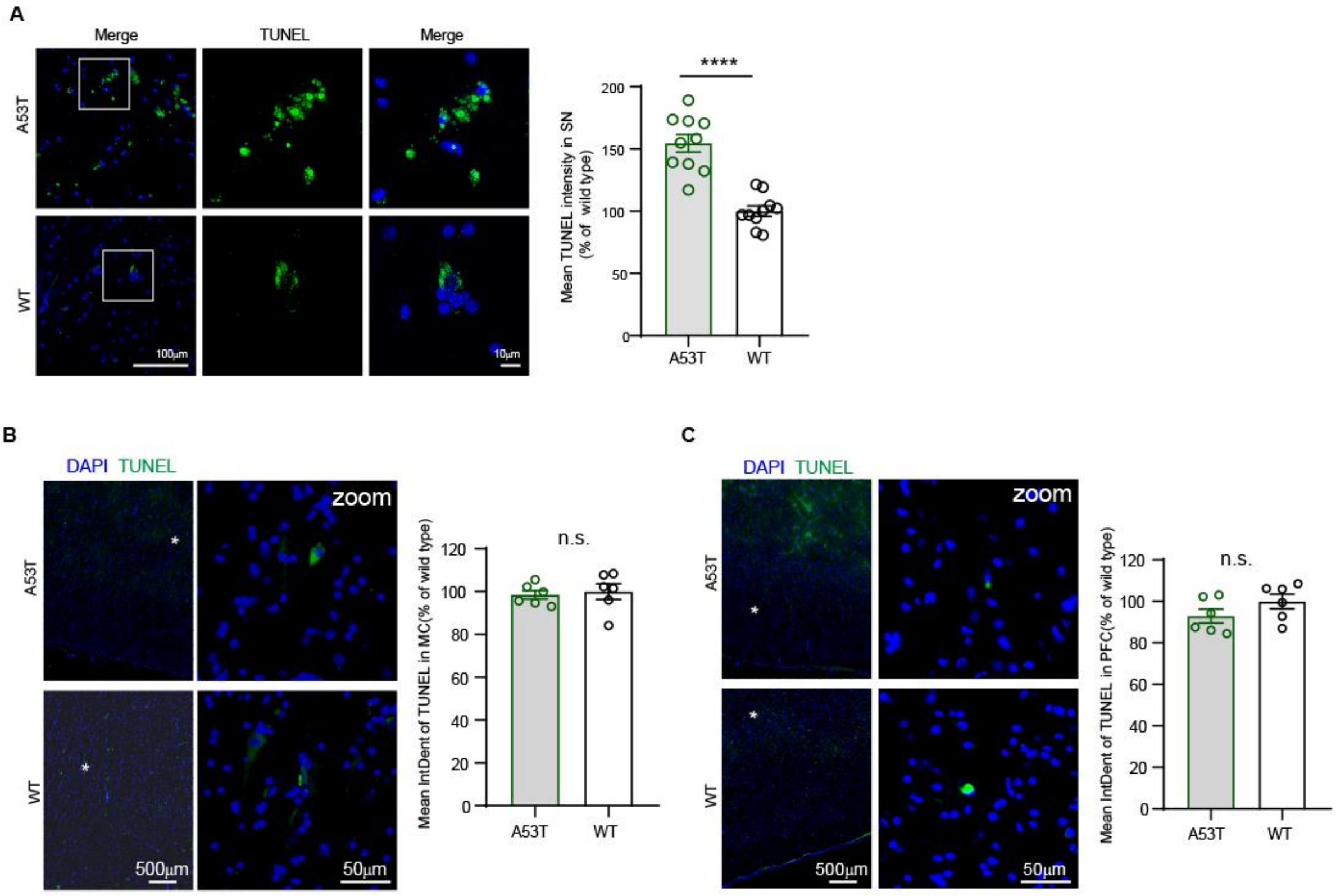

Supplementary Figure 4. TUNEL staining of A53T and WT monkey brains. (A) Representative micrographs (left) and quantification (right) of TUNEL level in the SN region. (B) Representative micrographs (left) and quantification (right) of TUNEL level in the motor cortex region. (C) Representative micrographs (left) and quantification (right) of TUNEL level in the prefrontal cortex region. Data are represented as mean ± SEM. *n* = 10 sections from 2 animals for each group in (A), *n* = 6 sections from 2 animals for each group in (B) and (C). Two-tailed Student's t-test and significance indicated as ****$p < 0.0001$.

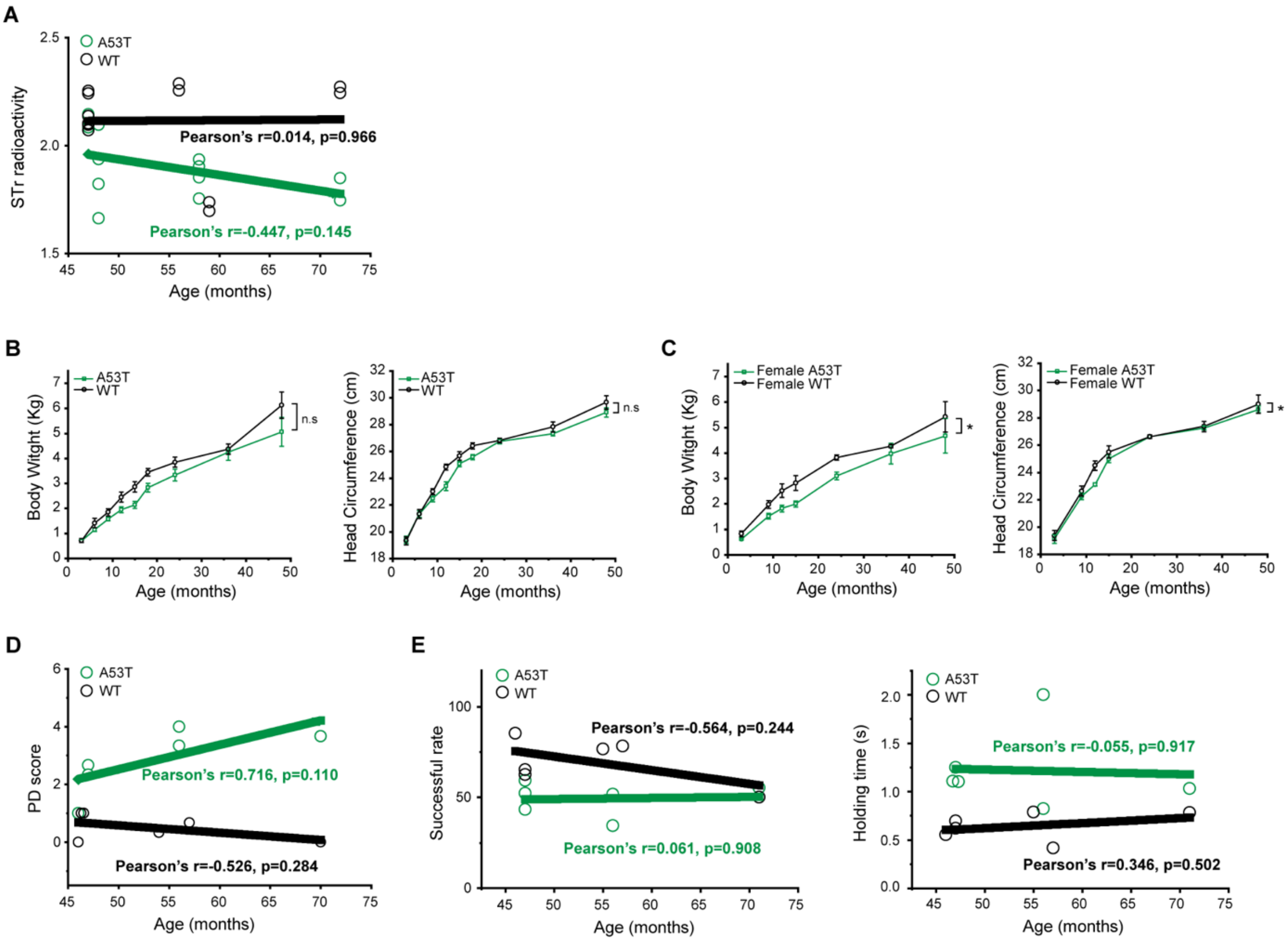

Supplementary Figure 5. Age-dependence of physical and behavioral tests. (A)The correlation between age and SPECT radioactivity. (B) Body weight (left) and head circumference (right) of TG and WT monkeys from 3 to 48 months (for body weight, *n* = 6 for both TG and WT monkeys, $P_{age} < 0.001$, $P_{genotypes} = 0.143$, $P_{interaction} = 0.270$; for head circumference, *n* = 6 for both TG and WT monkeys, $P_{age} < 0.001$, $P_{genotypes} = 0.212$, $P_{interaction} = 0.044$). (C) Body weight (left) and head circumference (right) of female TG and female WT monkeys used in MRI scans from 3 to 48 months (for body weight, *n* = 4 for both TG monkeys and WT monkeys, $P_{age} < 0.001$, $P_{genotypes} = 0.037$, $P_{interaction} = 0.472$; for head circumference, *n* = 4 for both TG monkeys and WT monkeys; $P_{age} < 0.001$, $P_{genotypes} = 0.024$, $P_{interaction} = 0.373$). (D) The correlation between age and PD score. (E) The correlation between age and finger fine motor function.

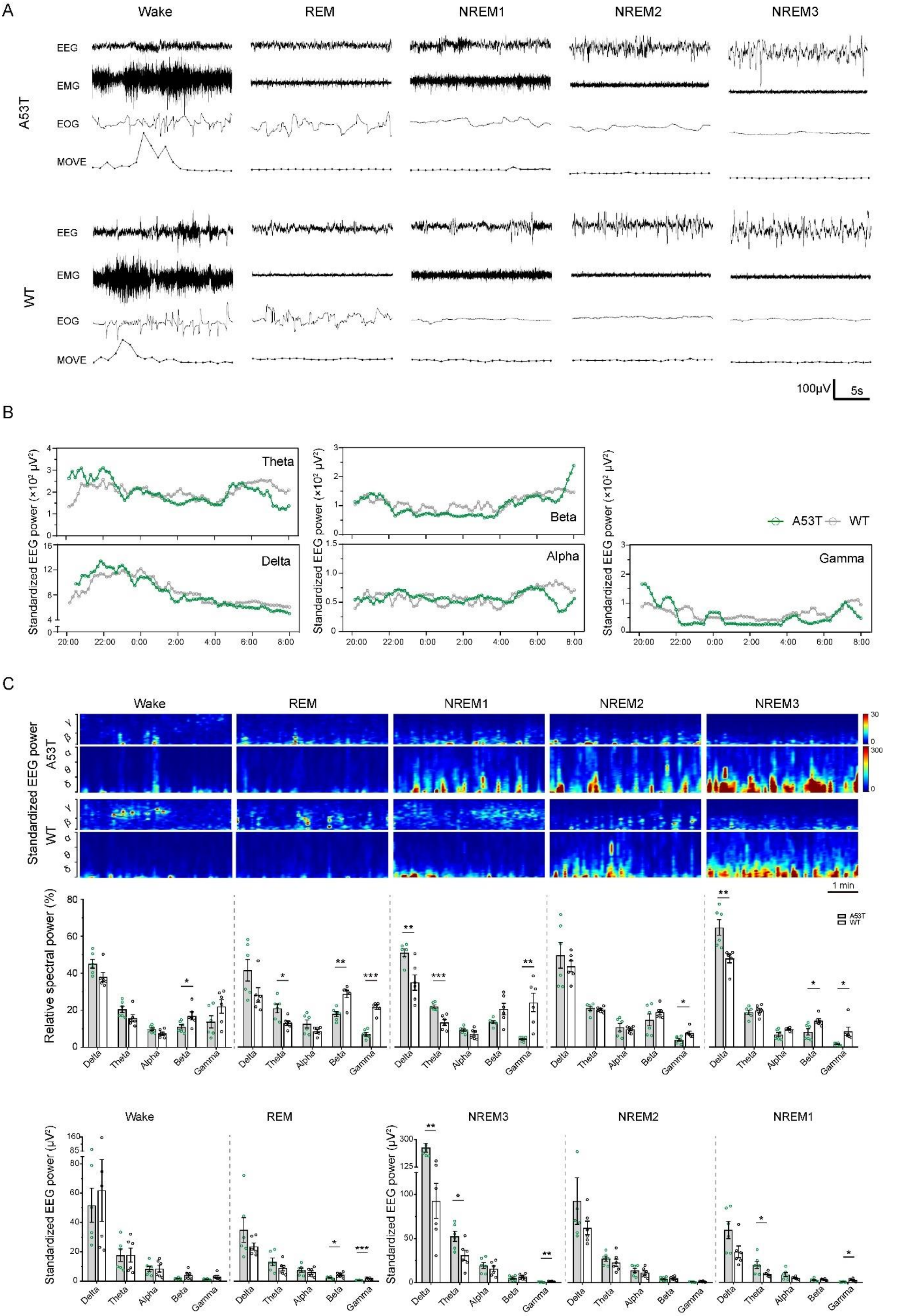

Supplementary Figure 6. Sleep behavioral test and polysomnography analysis reveal sleep disorder in A53T TG monkeys. (A) Representative traces of electroencephalogram (EEG), electromyogram

(EMG), electrooculogram (EOG) and movement for A53T TG (top) and WT monkeys (bottom). (B) Temporal distribution of EEG power spectra. (C) Representative spectrograms (top) and quantification of relative (middle) and standardized (bottom) contribution of distinct frequency bands during each vigilance states in A53T TG and WT monkeys. Data of 6 nights from 2 animals for each group are analyzed.

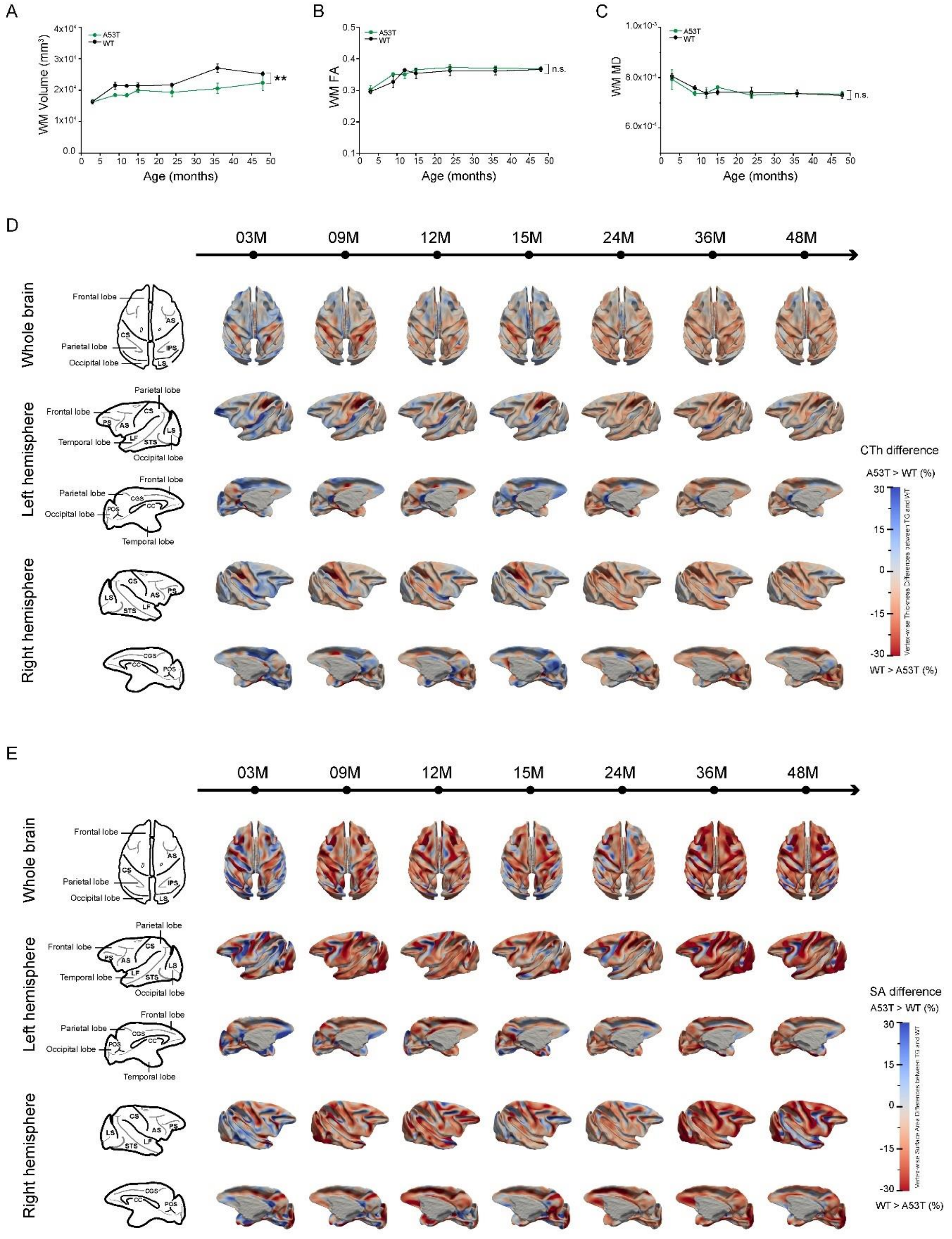

Supplementary Figure 7. Brain structural characteristics revealed by MRI. (A) The longitudinal changes by age in months of the volume of white matter (WM) for A53T and WT monkeys. (B) The longitudinal changes of GM (left) and WM (right) fractional anisotropy (FA) by age for A53T and WT monkeys. (C) The longitudinal changes of GM (left) and WM (right) mean diffusivity (MD) by age for A53T and WT monkeys. (D) False-colored cortical maps showing cortical thickness difference between TG and WT monkey brains. (E) False-colored cortical maps showing cortical surface area difference between TG and WT monkey brains.

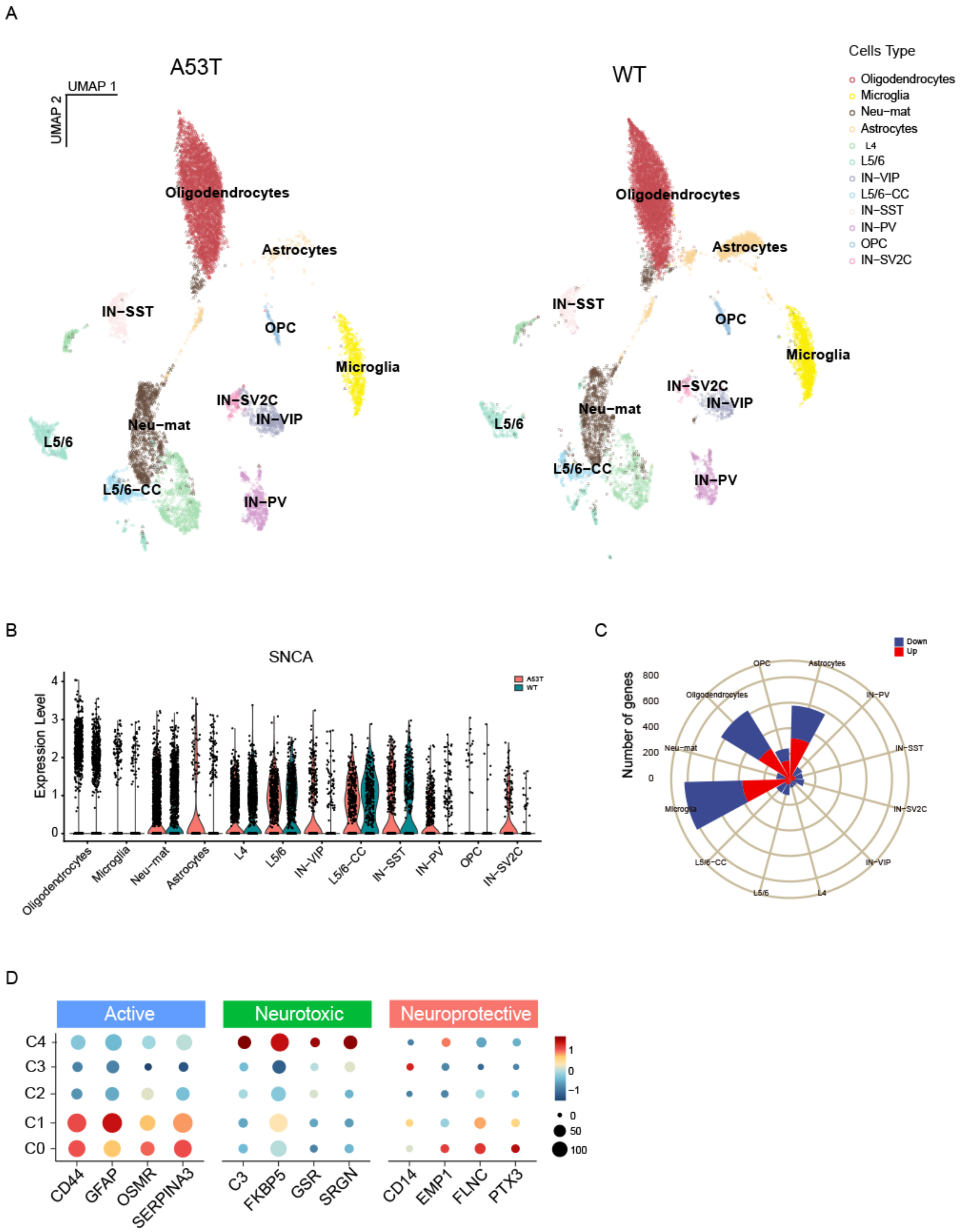

Supplementary Figure 8. DEGs and SNCA expression in A53T TG monkey brains revealed by snRNA-seq. (A) Contribution of nuclei from A53T TG (left) and WT (right) monkeys to each cell type. (B) Number of DEGs in different cell types. (C) Expression of SNCA in different cell types. (D) The expression level (as color scale) and the percent of cells expressing (as dot size) of marker genes for substates across clusters of astrocytes.

Supplementary tables

Supplementary Table 1. Animal Information.

Animal	Code of animals	Date of birth	Sex	Estimated *SNCA A53T* copy numbers*	MRI scanning	DAT SPECT	DA metabolites	Behavioral tests					Pathological analysis	Sleep EEG	Notes
								Parkinsonian motor score	Fine motor skills	Night-sleep analysis	Object discrimination	Working memory test			
**TG01**	NA	2011/04/09	F	2-3	-	-	-	-	-	-	-	-	-	-	Sacrificed after birth for Bulk RNA-seq
**TG02**	NA	2011/05/03	F	2	-	-	-	-	-	-	-	-	-	-	Sacrificed after birth for Bulk RNA-seq
**TG03**	110217	2011/05/09	M	2	-	Included	Included	Included	Included	Included	Included	Included	-	-	alive
**TG04**	120666	2012/06/13	F	1-2	Included	Included	Included	Included	Included	Included	Included	-**	Included	-	Sacrificed for pathological and scRNA tests
**TG05**	120676	2012/06/13	F	3-4	Included	Included	Included	Included	Included	Included	Included	Included	Included	-	Sacrificed for pathological and scRNA tests
**TG06**	130996	2013/03/29	F	5	Included	Included	Included	Included	Included	Included	Included	Included	-	Included	alive

**TG07**	130916	2013/04/02	F	7	Included	Included	Included	Included	Included	Included	Included	Included	-	Included	alive
**TG08**	130919	2013/04/22	M	2	-	Included	Included	Included	Included	Included	Included	Included	-	-	alive
**WT01**	NA	2011/05/04	F	-	-	-	-	-	-	-	-	-	-	-	Sacrificed after birth for Bulk RNA-seq
**WT02**	NA	2011/05/10	F	-	-	-	-	-	-	-	-	-	-	-	Sacrificed after birth for Bulk RNA-seq
**WT03**	110215	2011/05/10	M	-	-	Included	Included	Included	Included	Included	Included	Included	-	-	alive
**WT04**	121010	2012/05/12	F	-	Included	Included	Included	Included	Included	Included	Included	-**	-	-	alive
**WT05**	120528	2012/08/03	F	-	Included	Included	Included	Included	Included	Included	Included	Included	-	-	alive
**WT06**	130900	2013/04/07	F	-	Included	Included	Included	Included	Included	Included	Included	Included	-	Included	alive
**WT07**	130910	2013/04/15	F	-	Included	Included	Included	Included	Included	Included	Included	Included	-	Included	alive
**WT08**	130999	2013/04/25	M	-	-	Included	Included	Included	Included	Included	Included	Included	-	-	alive
**WT09**	151020	2015/05/29	F	-	Included			-	-	-	-	-	-	-	alive

**WT10**	150028	2015/06/28	F	-	Included			-	-	-	-	-	-	-	alive
**WT11**	140096	2014/04/30	F	-	Included			-	-	-	-	-	-	-	alive
**WT12**	141012	2014/05/13	F	-	Included	-	-	-	-	-	-	-	-	-	alive
**WT13**	141032	2014/07/29	F	-	Included	-	-	-	-	-	-	-	-	-	alive
**WT14**	141029	2014/04/17	M	-	-			-	-	-	-	-	Included	-	Sacrificed for pathological and scRNA tests
**WT15**	133118	2013/05/22	F	-	-			-	-	-	-	-	Included	-	Sacrificed for pathological and scRNA tests

*The copy numbers of *SNCA A53T* TG monkeys were estimated by Quantitative PCR, reported in ref 15.

**The TG02 did not meet the criteria for execution factors was excluded from the working memory test, the data of paired WT control of TG02 was also excluded.

Supplementary Table 2. The MRI data of female monkeys used for comparison of brain development.

**Animal**	**Ages (months)**						
	**Scan 1**	**Scan 2**	**Scan 3**	**Scan 4**	**Scan 5**	**Scan 6**	**Scan 7**
**TG04**	-	-	12.3	-	24.6	37.1	47.7
**TG05**	-	-	12.3	-	24.6	37.1	47.7
**TG06**	3.4	9.2	12.6	15.3	23.9	35.6	46.9
**TG07**	3.3	9.1	12.5	15.2	23.8	35.5	46.8
**WT04**	3.1	9.0		15.4	24.8	36.1	46.8
**WT05**	3.2	8.8		15.2	24.6	35.9	46.6
**WT06**	3.4	9.4	12.5	15.8	24.1	36.8	47.4
**WT07**	3.2	9.1	12.2	14.5	23.9	36.6	47.2
**WT09**	-	-	-	-	-	36.7	-
**WT10**	-	-	-	-	-	35.7	-
**WT11**	-	-	-	-	-	-	49.8
**WT12**	-	-	-	-	-	-	49.4
**WT13**	-	-	-	-	-	-	46.8

Supplementary Table 3. The regional GM volume changes between TG and WT monkeys.

Location	Brain subdivisions	*p* of GM volume difference between TG and WT monkeys													
		R							L						
		3	9	12	15	24	36	48	3	9	12	15	24	36	48
**Cortical**															
Frontal	Superior frontal gyrus	0.800	0.800	0.533	0.800	0.343	0.073	0.024*	0.800	0.267	0.533	0.800	0.200	0.073	0.042
	Middle frontal gyrus	1.000	0.267	0.267	0.800	0.486	0.006*	0.042	1.000	0.133	0.267	0.533	0.486	0.042	0.109
	Inferior frontal gyrus	0.800	0.133	0.267	0.533	0.343	0.012*	0.23	1.000	0.267	0.267	0.267	0.343	0.012*	0.073
	Frontal orbital gyrus	0.133	0.133	0.533	1.000	0.486	0.315	0.315	1.000	0.133	0.133	0.800	0.343	0.073	0.109
	Medial orbital gyrus	0.533	0.800	0.133	1.000	0.343	0.042	0.109	0.133	0.533	0.533	0.533	0.029	0.109	0.23
	Lateral orbital gyrus	0.800	0.533	0.267	1.000	0.486	0.23	0.315	1.000	0.267	0.267	0.533	0.486	0.164	0.315
	Straight gyrus	0.533	1.000	0.267	0.800	0.200	0.164	0.927	0.533	0.800	0.267	0.533	0.200	0.164	0.23
	Insula	0.133	0.267	1.000	0.133	1.000	1	0.927	0.133	0.267	1.000	0.267	0.486	0.927	0.23
Parietal	Precentral gyrus	0.533	0.533	0.533	0.533	0.114	0.073	0.024*	0.533	0.533	0.533	0.133	0.114	0.042	0.024*
	Postcentral gyrus	0.800	0.133	0.133	0.133	0.114	0.012*	0.012*	0.800	0.133	0.133	0.133	0.057	0.012*	0.012*
	Superior parietal lobule	0.800	0.133	0.133	0.133	0.057	0.042	0.024*	1.000	0.267	0.267	0.133	0.057	0.012*	0.024*
	Supramarginal gyrus	0.800	0.133	0.133	0.267	0.029	0.012*	0.024*	0.533	0.133	0.133	0.133	0.029	0.006*	0.006*
	Angular gyrus	0.800	0.133	0.133	0.533	0.114	0.006*	0.006*	0.267	0.133	0.133	0.133	0.029	0.006*	0.006*
	Annectant gyrus	0.800	0.133	0.133	0.533	0.029	0.024*	0.024*	0.800	0.133	0.133	0.267	0.029	0.006*	0.012*
Temporal	Superior temporal	0.133	0.267	0.267	0.800	0.114	0.012*	0.109	0.267	0.533	0.533	0.800	0.200	0.012*	0.073
	Middle temporal gyrus	1.000	0.133	0.133	0.267	0.200	0.012*	0.024*	1.000	0.133	0.133	0.267	0.057	0.012*	0.024*
	Inferior temporal gyrus	0.800	0.133	0.133	0.267	0.200	0.006*	0.006*	0.267	0.133	0.133	0.533	0.057	0.024*	0.024*
	Entorhinal area	0.533	0.267	0.800	0.800	0.686	0.042	0.315	0.800	0.267	0.533	0.133	0.686	0.042	0.006*
	Parahippocampal gyrus	0.800	0.133	1.000	1.000	0.686	0.042	0.315	0.800	0.133	0.133	0.800	0.886	0.012*	0.109
	Fusiform gyrus	0.800	0.133	0.800	0.800	0.886	0.024*	0.073	0.133	0.133	1.000	0.800	0.029	0.012*	0.164
	Lingual gyrus	0.133	0.533	0.533	0.133	0.343	0.412	0.315	0.267	0.533	0.800	0.133	0.343	0.23	0.788
Occipital	Occipital gyrus	0.800	0.533	0.800	0.800	0.343	0.073	0.109	1.000	0.533	0.533	1.000	0.343	0.109	0.042
	Inferior occipital gyrus	0.267	0.800	0.267	0.800	0.886	0.109	0.23	0.133	0.533	0.800	0.533	0.686	0.073	0.042
Medial	Splenium of the corpus	1.000	0.533	0.533	0.267	0.686	0.648	0.927	0.800	0.267	0.800	0.267	0.686	0.927	0.927
	Body of the corpus	1.000	0.533	0.533	0.800	1.000	0.315	0.788	0.267	0.533	0.800	1.000	0.686	0.073	0.927

	Genu of the corpus	0.133	0.133	0.267	0.533	0.886	0.412	0.164	0.533	0.533	1.000	1.000	0.686	0.927	1
	Anterior cingulate gyrus	0.533	1.000	0.533	0.533	0.886	0.012	0.073	0.533	0.133	0.133	0.800	0.486	0.073	0.109
	Posterior cingulate	0.133	0.133	1.000	0.533	0.886	0.648	0.648	0.533	0.800	0.800	1.000	0.486	0.073	0.527
	Isthmus of the	0.133	0.533	0.533	0.533	0.886	0.648	0.527	0.533	0.800	0.533	0.800	0.686	0.927	0.164
	Precuneus	0.533	0.533	0.267	0.800	0.686	0.042	0.073	0.533	0.533	0.133	0.267	0.686	0.073	0.164
	Cuneus	0.267	0.267	0.267	0.800	0.343	0.042	0.024	0.533	1.000	0.533	0.533	0.200	0.109	0.073
**Subcortical**	Caudate	0.800	0.133	0.133	0.533	0.029	0.010	0.012	0.267	0.133	0.133	0.533	0.029	0.010	0.012
	Putamen	0.533	1.000	0.267	0.800	0.343	0.476	0.527	0.267	0.800	0.267	1.000	0.343	0.352	0.648
	Hippocampus	0.800	0.133	0.133	0.800	1.000	0.171	0.527	1.000	0.133	0.133	0.267	0.686	0.171	0.109
	Amygdala	0.800	0.800	0.133	0.533	0.057	0.019	0.073	0.800	0.800	0.133	0.533	0.114	0.038	0.073

Comparisons of regional GM volume between the A53T and WT control groups. The subdivisions were presented when the GM volume of it was statistical significance at stage of 7 time point by Mann-Whitney U Test. Considering that the statistical significance of the difference appeared in 2 stages, the significance threshold was set as $p < 0.025$ to reduce the type I error. L, left hemisphere; R, right hemisphere.
